# Mass Spectrometric Determination of Site-Specific *O*‑Acetylation in Rhamnogalacturonan I Oligomers

**DOI:** 10.1021/jasms.5c00440

**Published:** 2026-03-30

**Authors:** Liyanage Devthilini Fernando, Xu Yang, Stephanie Archer-Hartmann, Lubana Shahin, Liang Zhang, Breeanna R. Urbanowicz, Christian Heiss, Parastoo Azadi

**Affiliations:** Complex Carbohydrate Research Center, 123423 University of Georgia, Athens, Georgia 30602, United States

**Keywords:** RG-I, pectin, oligosaccharide, *O*-acetylation, mass spectroscopy, tandem MS, derivatization

## Abstract

*O*-acetylation, a common modification in rhamnogalacturonan I (RG-I), is critical for various biological processes, including plant growth, stress responses, and pathogen defense. Precise determination of the degree and specific positions of acetylation is therefore essential. To date, nuclear magnetic resonance (NMR) and tandem mass spectrometry have been employed to identify *O*-acetyl positions in pectin oligosaccharides. Although NMR is effective, it requires pure, high-concentration samples. Tandem mass spectrometry (MS), which uses smaller sample amounts, faces challenges due to *O*-acetyl migration between monosaccharide positions. The multiple steps in pectin sample analysis can further promote *O*-acetyl migration, especially near free hydroxyl groups. Moreover, during tandem MS, *O*-acetyl groups may detach, complicating the accurate tracking. This study presents an approach to lock *O*-acetyl groups by introducing trideuteroacetyl and propionyl substituents onto free hydroxyls of RG-I or partially acetylated RG-I. By combining matrix-assisted laser desorption/ionization time-of-flight (MALDI-TOF) MS and electrospray ionization (ESI) MS with MS/MS or tandem mass spectrometry (MS^n^), we devised a way to determine the monosaccharide sequence in the oligomer and the precise positions of *O*-acetyl groups in partially acetylated RG-I. This method enables the study of the regiospecificity of recombinant pectin *O*-acetyltransferases and can be applied to other oligosaccharides to determine acyl positions.

## Graphical abstract

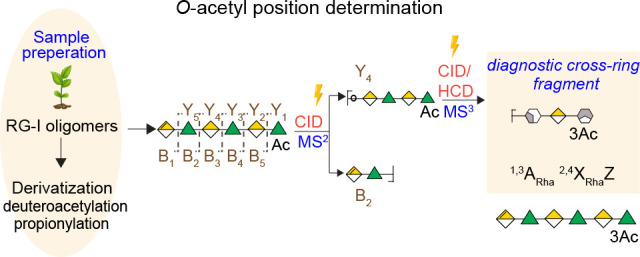

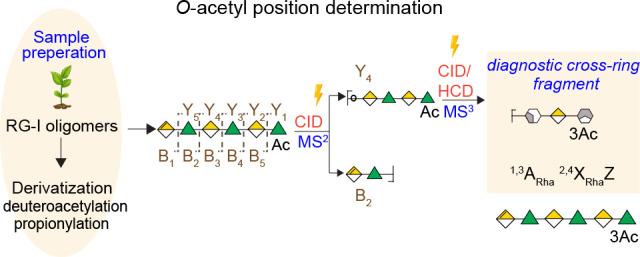

## Introduction

Rhamnogalacturonan I (RG-I) is a structurally complex pectic polysaccharide that is present in the cell walls of all vascular plants.^1–3^ Due to its structural complexity, little is known about RG-I structural diversity in different cells, tissues, and species or about how it interacts with cellulose and other polysaccharides to regulate the properties and functions of the cell wall. *O*-Acetylation (*O*Ac) is a common noncarbohydrate modification observed in pectin RG-I and plays crucial roles in various biological processes such as growth of plants,^4,5^ abiotic stress responses,^6^ and defense pathways against pathogens.^7^ To study these processes on a molecular level, it is essential to accurately determine both the overall degree of *O*-acetylation and the specific positions of the *O*-acetyl groups.

To date, both nuclear magnetic resonance (NMR) and tandem mass spectroscopy (MS^n^) have been employed to identify the acetylated positions in the pectin oligosaccharides.^8–18^ While NMR is a powerful tool, it requires pure samples and high concentrations. In contrast, tandem mass spectroscopy (MS^n^) can precisely locate acetylation and methylation in oligogalacturonides derived from enzymatic hydrolysis of pectin using very low sample amounts (5 μg).^15–17^ Nevertheless, this task is hampered due to the tendency of *O*-acetyl groups to migrate between different positions on a given monosaccharide residue,^8,19^ multiple isolation and analysis steps involved in studying pectin samples provide ample opportunities for *O*-acetyl migration, particularly in the presence of nearby free hydroxyl groups.^19–22^ Further, when performing tandem MS, *O*-acetyl groups can be lost, preventing the detection of a diagnostic fragment and hindering accurate position tracking.^23–25^

In previous studies, several attempts have been made to prevent migration of *O*-acetyls and to determine their location in the oligosaccharides by derivatizing the sample using propionylation,^22^ trideuteroacetylation,^26^ and neutral permethylation.^27^ Propionylation has been applied to starch acetates, chitin, and cellulose esters for structural studies by NMR spectroscopy.^28–30^ Further, combined propionylation and deuteroacetylation approaches were employed for unambiguous identification of naturally occurring modifications in sialic acid from biological sources using fast atom bombardment-mass spectroscopy (FAB-MS).^26^ Reinhold et al. used neutral permethylation to locate the succinyl and acetyl position in exopolysaccharide succinoglycan from *Rhizobium meliloti* by collision-induced dissociation (CID) fragmentation in tandem mass spectroscopy.^27^ Similarly, the precise location of acetyl groups in the core oligosaccharide of *Rhizobium trifolii* was determined using perdeuteroacetylated derivatives analyzed by FAB-MS.^31^ Sharp et al. also demonstrated that CID could effectively determine the position of *O*-acetyl groups in partially methylated and partially acetylated chondroitin sulfate and heparan sulfate oligosaccharides.^32,33^

Previous studies have demonstrated that chemical derivatization not only fixes the *O*-acetyl groups but also enhances the ionization efficiency while lowering the detection limit, particularly in higher-order MS^n^ (*n* > 2) experiments, and it also shifts analyte masses into regions of reduced chemical noise, enabling more confident detection of low-abundance fragments.^34^ Despite these advantages, most mass spectrometric studies on RG-I oligosaccharides have focused on nonderivatized species and primarily employed CID, which typically yields dominant glycosidic cleavages and provides limited information on branching patterns or *O*-acetylation sites.^35–38^ Although several reports have addressed the sequencing and general structural features of RG-I oligomers, detailed mass spectroscopic investigation focusing on the precise determination of *O*-acetylation sites in the pectin oligosaccharides, especially those derived from RG-I remain scarce.

In the present study, we address this limitation by applying the established derivatization strategies, trideuteroacetylation and propionylation, to partially *O*-acetylated RG-I oligomers to effectively lock the natively present *O*-acetyl groups while derivatizing free hydroxyls. Building on earlier methodologies, we combine derivatization with both CID and higher-energy collision dissociation (HCD) to promote enhanced cross-ring fragmentation, enabling confident assignment of *O*-acetylation sites in RG-I. To our knowledge, these derivatization approaches and the combined CID/HCD fragmentation patterns have not previously been applied to RG-I oligomers. Integrating matrix-assisted laser desorption/ionization time-of-flight mass spectroscopy (MALDI-TOF MS), electrospray ionization mass spectroscopy (ESI-MS) with MS/MS or MS^n^ tandem mass capabilities, and liquid chromatography (LC) separation with MS^n^ schemes allows us to determine the structure of the oligomers and the precise positions of *O*-acetyl groups in partially acetylated RG-I. Specifically, this analytical flow helped to get more structural insight into the *O*-acetyl positions of trichome51 birefringence 3-*O*-acetyl transferase (TBR) enzyme acetylated RG-I oligomers^37^ and the native celery RG-I oligomers than previous methodological reports.^37,38^ This method provides a robust platform for probing the regiospecificity of recombinant pectin *O*-acetyltransferases and is readily extendable to other plant oligosaccharides to determine the acyl positions.

## Materials and Methodology

Acetic anhydride-d_6_ (99 atom %D) was obtained from Sigma-Aldrich (St. Louis, MO, USA). Pyridine was purchased from J.T Baker (Phillipsburg, NJ, USA) and propionic anhydride was purchased from Sigma-Aldrich. Dichloromethane (DCM), used for the extraction of the derivatized sample, was purchased from Sigma-Aldrich. The 2,5-dihydroxybenzoic acid (DHB) matrix was purchased from Sigma-Aldrich, MO, USA. The ESI-MS solvent acetonitrile (ACN) was purchased from Honeywell (Tokyo, Japan). RG-I (*Arabidopsis mucilage*) partially acetylated oligosaccharide and digested RG-I celery samples were obtained from the laboratory of Dr. Breeanna Urbanowicz at the Complex Carbohydrate Research Center, University of Georgia.

### Sample Preparation

#### Enzymatically *O*-acetylated RG-I Degree of Polymerization 6 (DP6)

Pectin lyase digested *A. mucilage* RG-I oligomers (average degree of polymerization 6) were enzymatically acetylated using trichome51 birefringence (TBR) enzyme using 4-methylumbelliferyl acetate as the donor in the MES-buffer at 30 °C, as reported previously.^37^ The acetylated RG-I product was separated using a Superdex peptide 300/10 GL column (Cytiva) connected to an Agilent 1260 Infinity II high-performance liquid chromatography system (HPLC). 10 mM ammonium acetate was used as the mobile phase at a 0.5 mL/min rate. The RG-I containing fractions were lyophilized and further analyzed with NMR and mass spectroscopy.

#### Native Celery RG-I

The celery RG-I oligomers were prepared using the BT4175 RG-I lyase enzyme as reported previously.^38^ It cleaves the backbone and generates an unsaturated double bond between C4 and the C5 of the nonreducing GalA residues in the RG-I backbone.^38^ The digested celery RG-I oligomers were directly used for derivatization and analysis.

### Derivatization

The peracetylation procedure was modified from Xu et al.^28^ RG-I pyridine and acetic anhydride-d6 (1:1) were reacted at 60 °C for 24 h. The propionylation was carried out in the same way with pyridine and propionic anhydride (1:1) at 60 °C for 24 h. The reaction product was extracted with DCM and washed five times with water. The DCM layer was dried under dry air. The samples were dissolved in acetonitrile prior to mass spectroscopy analysis.

### MALDI-TOF

Samples were dissolved in an acetonitrile or methanol–water 1:1 mixture (∼final concentration 20 mg/mL) for MALDI-TOF analysis. 1 μL of sample was spotted on a ground steel MALDI plate with 1 μL of 2,5-dihydroxybenzoic acid (DHB) matrix (15 mg/mL; in 70% ACN:0.1% formic acid), and after crystallization, the spot was analyzed on a Bruker rapfleX Tissuetyper MALDI-TOF MS instrument (Bruker Daltonics, Germany) under reflector mode in positive or negative ionization mode. The mass spectra were processed for smoothing and baseline subtraction using FlexAnalysis version 3.4 (Bruker Daltonics, Germany). For MS^2^ analysis on the MALDI-TOF instrument, the targeted ions were isolated and subjected to collision-induced dissociation (CID) for fragmentation analysis.

### Direct Infusion (DI) ESI-MS^n^ Analysis of RG-I DP6

Experiments utilizing direct infusion (DI) ESI-MS^n^ on derivatized RG-I oligosaccharides were performed on an Orbitrap Eclipse Tribrid mass spectrometer with both positive and negative polarities as needed.

Derivatized sample solutions were diluted to 20–50 μg/mL with acetonitrile. Direct infusion was conducted at a flow rate of 3 μL/min. The EASY-Max NG heated ESI (H-ESI) source used in the experiments was supplied by nitrogen generators. A HESI-II probe with a low-flow needle insert was used for generating highly sensitive MS^n^ spectra. The ion transfer tube was maintained at 325 °C, and the vaporizer was held at 52 °C. The spray voltage and aux gas, sheath gas, and sweep gas flow rates were adjusted for optimal signal intensity and stability. For precursor isolation, a quadrupole was used, while the radio frequency (RF) lens is the most critical factor directing the enhancement of ions at different mass ranges. Most precursor ions of different levels of MS^n^ were able to be fragmented by 30–45% (normalized collision energy) CID. However, some of the derivatized RG-I fragments were resistant to 100% CID fragmentations. Further higher-energy collision dissociation (HCD) was used for MS^3^ fragmentation to obtain cross-ring fragmentation. A total of 100 scans were averaged for each experiment to improve the data quality.

### LC-ESI-MS^n^ Analysis of Celery RG-I

A derivatized RG-I sample from celery was subjected to LC-MS^n^ analysis on an Ultimate 3000 RSLCnano system coupled with an Orbitrap Fusion Tribrid mass spectrometer (Thermo Scientific). A Commercial Acclaim Pepmap100 C18 0.75 mm × 150 mm column operated at 55 °C was used for sample separation. Buffer A was 0.1% formic acid; Buffer B was 0.1% formic acid in 80% acetonitrile. The elution gradient was 10–95% B in 40 min after the column was equilibrated by 10% B for 10 min at the beginning of each injection. Ten microliters of the sample were injected. The targeted MS^3^ spectra were recorded in parallel with a data-dependent acquisition MS^2^ program for precursor screening. A 3 Da isolation window was applied for 45% CID MS^2^, and a 4 Da isolation window was applied for 35% HCD MS^3^. Quadrupole isolation was used, and the RF lens was set at 60%. The target MS^n^ list was prepared based on direct infusion observations and theoretical masses calculated using GlycoWorkbench 2.1 and ChemDraw 23.0.

### Q Exactive Analysis of Celery RG-I

The native RG-I celery sample was also analyzed using a Vanquish Ultra Performance LC (UHPLC) (Thermo Fisher Scientific) connected to a Thermo Q Exactive HF Orbitrap mass spectrometer (Thermo Fisher Scientific). The separation was carried out on a commercial C18 column (Zorbax Eclipse XDB-504C18; 2.1 mm × 150 mm; 1.8 μm) with a linear gradient from 1% to 99% acetonitrile in water containing 0.1% formic acid at a flow rate of 0.3 mL/min for 22 min. The column was cleaned for 2 min with 99% acetonitrile, followed by 4 min of reconditioning with 1% acetonitrile. A data-dependent program was used for acquisition in the positive ion mode, where the precursor ion scan (MS^1^) was acquired at 120 k resolution from 200 to 2000, followed by top-down fragmentation (stepped HCD; MS^2^) of high-to-low intensity m/zs at 30 k resolution.

### NMR Analysis of RG-I

Approximately ∼0.5–1 mg of the partially *O*-acetylated RG-I DP6 oligomer and RG-I celery was dissolved in 200 μL of D_2_O (99.9% D) and lyophilized overnight. Then, the sample was dissolved in 40 μL of D_2_O (99.96% D). The dissolved sample was transferred to a 1.7 mm NMR tube for NMR analysis. NMR data were acquired at 25 °C on a Bruker Avance NEO spectrometer (^1^H, 800 MHz) equipped with a cryoprobe using standard pulse sequences. The acquisition parameters are tabulated below in Supporting Table S1. Chemical shifts were referenced to 3-(trimethylsilyl)-propanesulfonic acid sodium salt (DSS-*d*_6_) peak (δ_H_ = 0.0 ppm, δ_C_ = 0.0 ppm), for RG-I DP6 oligomer and for RG-I celery, referenced to HOD peak (δ_H_ = 4.78 ppm). The spectra were processed and analyzed with Bruker TopSpin 4.1.3 and *MestReNova* v14.2.1–27684.

### Data Analysis

The general method for *O*-acetylation position determination is summarized in Supporting Methods S1. The ESI-MS^n^ data obtained from the Orbitrap Tribrid mass spectrometer were analyzed using Freestyle software version 1.8 SP1­(Thermo Fisher Scientific). The MALDI-TOF MS/MS spectra were analyzed using Bruker flex ABakysis (version 4.2). The fragmentation patterns were predicted using the GlycoWorkbench (version 2.1) for native and deuteroacetylated RG-I, and ChemDraw (version 23.0.1.10) was used to predict the propionylated RG-I fragmentation.

## Results and Discussion

### Derivatization of RG-I

To develop a robust method for determining the *O*-acetyl position in RG-I, the first step was to find the best way to derivatize the samples for the purpose of blocking all free hydroxyls to prevent migration of the *O*-acetyl groups. Initially, we carried out derivatization using different acylating reagents on neutral (maltohexose) and acidic (RG-I) oligomers to find the optimum conditions to get fully acylated oligomers. We used slightly modified reported Methods **1–3** (Table 1) either to acetylate or to propionylate the oligomers. Method **2** gave fully derivatized neutral and RG-I oligomers, but Method **1** yielded incomplete derivatization (Supporting Figure S1). Method **3** (perchloric acid) was too harsh even for neutral oligomers and led to sample degradation (Supporting Figure S1). The pyridine catalyst (in Method **2**) typically allows for more selective and milder conditions and neutralizes the byproducts^39^ and was also used previously to acylate pectin.^40,41^ Therefore, we used pyridine with the appropriate acid anhydride as the acylating reagent in this study.

**Table 1:** Acylating Reagents Used for Derivatization

method	acylating reagent	reaction conditions	refs	outcome
**1**	trifluoracetic anhydride and appropriate acid (propionic acid, deuteroacetic acid)	24 h at 60 °C	Manzi et al.^26^	partial derivatization
**2**	pyridine and propionic anhydride/acetic anhydride	24 h at 60 °C	Xu et al.^28^	fully derivatized
**3**	propionic anhydride/perchloric acid	2 h at room temperature	Zhong et al.^29^	too harsh
**4**	methyl trifluoromethanesulfonate in trimethyl phosphate	2 h at 50 °C	Prehm et al.^42^	not successful

We applied the pyridine trideuteroacetic anhydride/propionic anhydride derivatization condition (Method **2**) to the native unsaturated RG-I oligomer (unsaturated uronic acid residue at the nonreducing end of the oligomer) with a degree of polymerization of 6 (DP6, molecular weight *M*_W_ = 966 Da) obtained from *A. mucilage* by lyase digestion (without *O*-acetyl groups). This method gave full acylation of the RG-I oligomer (Supporting Figure S2). The acylation reaction used a 1:1 mixture of pyridine:acetic anhydride-d_6_ or propionic anhydride at no more than 60 °C to prevent decomposition of the RG-I molecule. The reaction product was then extracted using dichloromethane, as the acylated RG-I is more hydrophobic than native RG-I. Based on the MALDI-TOF MS spectra, the product of the acylation of RG-I DP6 was 80–90% deuteroacetylated or propionylated. The acyl groups substituted all of the 2-OH and 3-OH of galacturonic acid (GalA) and 3-OH and 4-OH of rhamnose (Rha) in the RG-I oligomer.

We also attempted methylation of RG-I oligomers by Method **4**, using methyl trifluoromethanesulfonate in trimethyl phosphate, as reported in Prehm et al. 1980 ^42^ (Supporting Figure S3). However, this reaction condition was ineffective for the RG-I oligosaccharides, likely because of the limited solubility of acidic oligosaccharides in trimethyl phosphate. The lower pH and presence of moisture coming from the reagents may also have played a role in preventing methylation.

Based on the MALDI-TOF MS and MS/MS of the deuteroacetylated and propionylated samples, the respective singly charged [M + Na] ^+^ parent ion (*m*/*z* = 1574 and 1717) yielded, upon collision, numerous daughter ions characteristic of these oligosaccharides (Supporting Figure S2). The major ions are the result of B and Y cleavages, providing information related to the monosaccharide sequence of the RG-I oligosaccharide. The conversion of the RG-I oligosaccharides to their corresponding derivatives improved the ionization efficiency and detection sensitivity by 1 to 2 orders of magnitude compared with the detection of nonderivatized analytes and allowed for detection in lower concentrations (e.g., nanomolar, picomolar levels). When compared to native RG-I, deuteroacetylation increases the mass by 45 Da per acyl group, and propionylation increases the mass by 54 Da per acyl group. This derivatization was reproducible and consistently yielded derivatized DP6, DP8, and DP10 RG-I (Supporting Figure S4).

### Derivatization of Partially *O*-Acetylated RG-I and Analysis by MALDI-TOF-MS

Next, we exposed enzymatically partially (mono, di) *O*-acetylated RG-I DP6 molecules to the acylating reagents (deuteroacetic anhydride/propionic anhydride + pyridine) and performed MALDI-TOF-MS on peracylated samples (Supporting Figure 5A,C).

To determine the specific residue where *O*-acetylation occurs, we acquired MS^2^ spectra of the deuteroacetylated monoacetylated RG-I DP6 (RG-I *O*Ac) at (*m*/*z* = 1571) and the nonacetylated RG-I DP6 (*m*/*z* = 1574) using a narrow precursor isolation window (1–2 Da) to enhance specificity and minimize the contribution of chimeric spectra. The fragment ions are shown according to the Domon and Costello nomenclature^43^ for RG-I oligomers (Supporting Figure S5). The presence of B_3_ (*m*/*z* = 773) ion, in both the MS^2^ spectra (Supporting Figure S5B), suggests that the three residues from the nonreducing end are not *O*-acetylated. Likewise, the *m*/*z* = 839 ion was observed in the MS^2^ spectra of the propionylated derivatives of RG-I_*O*Ac and RG-I (non-*O*Ac) (Supporting Figure S5D).

The B_5_ daughter ions *m*/*z* 1275 in the deuteroacetylated and *m*/*z* = 1175 propionylated MS^2^ spectra suggested that acetylation is present on the reducing end of rhamnose. However, the *m*/*z* = 1272 B_5_ ion in the deuteroacetylated DP6 RG-I_*O*Ac (Supporting Figure S5B) suggests that the *O*-acetylation can also be either the second rhamnose or the first galacturonic acid residue from the reducing end.

Further pinpointing the exact position of *O*-acetylation was not feasible due to the instrument’s lack of tandem MS capability and the absence or low abundance of cross-ring fragments in MALDI-TOF/MS/MS spectra, which were attributed to the insufficient sensitivity provided by the instrument, even under CID conditions, to produce these specific fragmentation patterns.

### Finding the *O*-Acetyl Position of Enzymatically Monoacetylated RG-I Using Direct Infusion (DI) ESI-MS^n^

To find the exact position of the O-acetyl group in RG-I, we used the RG-I DP6 from *A. mucilage* that was partially acetylated using trichome51 birefringence 3-*O*-acetyl transferase (TBR) enzyme.^37^ The position of the *O*-acetyl group thus introduced into the native RG-I was analyzed by nuclear magnetic resonance spectroscopy (NMR) prior to derivatization and MS analysis. The 2D ^1^H–^1^H correlation spectroscopy (COSY) and ^1^H–^13^C heteronuclear single quantum coherence (HSQC) spectra obtained on the native monoacetylated RG-I DP6 (Supporting Figure S6 and Table S2) showed that the RG-I is acetylated only in the 3-*O* position of rhamnose. The *O*-acetylation was located at the reducing-end rhamnose and at the second rhamnose (third residue/monomer from the reducing-end), which was consistent with the reported specificity of the TBR enzyme.^37^ The NMR data showed about equal acetylation of the reducing-end and second rhamnose residues, but we could not determine if there were two equimolar monoacetylated RG-I oligomers only, a diacetylated RG-I oligomer only, or if there was a mixture of all three species. Then, the partially *O*-acetylated RG-I was derivatized (deuteroacetylated or propionylated) and further analyzed using electrospray ionization mass spectroscopy (ESI-MS).

ESI-MS allows tandem mass spectroscopy with higher-order MS and is equipped with different fragmentation modes, such as collision-induced dissociation (CID) and higher-energy collisional dissociation (HCD). This makes ESI-MS the ideal instrumentation for the structural analysis of RG-I oligosaccharides.

ESI-MS relies on the efficient desolvation of analytes to generate gas-phase ions. In this process, a high electric field disperses the analyte solution into fine microdroplets. As the solvent evaporates, ions are released and introduced into the mass spectrometer for analysis. Successful ionization depends not only on desolvation but also on the analyte’s solubility in the solvent system. After derivatization with deuteroacetyl and propionyl groups, the sample became more hydrophobic and showed less solubility in methanol (50–90%), which is the most common ESI-MS solvent. Therefore, we used 100% acetonitrile, which is a polar aprotic solvent with low hydrogen-bonding capability, as the solvent to solubilize the derivatized RG-I samples for ESI-MS analysis.

For the direct infusion (DI) ESI-MS analysis, we employed both collision-induced dissociation (CID) and higher-energy collision dissociation (HCD) MS^n^ at several collision energies to produce higher-quality MS^n^ spectra for improved fragment identification. In agreement with prior findings,^44^ we observed that CID resulted mostly in glycosidic cleavages;^44^ fragmentation across the sugar rings (cross-ring fragmentation) was less compared to HCD. This is because cross-ring cleavage requires the simultaneous rearrangement and breakage of two covalent bonds, which is intrinsically less favorable under CID conditions. In the ion trap, CID typically involves a longer series of low-energy collisions with the inert gas, and these often favor low-energy fragmentation pathways (glycosidic cleavages), suppressing the detection of labile cross-ring fragments.^45^

In contrast, HCD involves higher-energy collisions in a short time frame in ion routing modules. This higher energy imparts more internal energy to the precursor ions, increasing the probability of a wider range of sequential fragments and increasing the chances of forming more cross-ring fragments, and the produced fragments were preserved and sent for the high-resolution mass analyzer before quenching.^45^ Therefore, we generated MS^1^ and MS^2^ parent ions using CID with a wide isolation window to enrich glycosidic cleavage patterns, but acquired MS^3^ spectra using HCD to enhance the detection of cross-ring fragments.

#### Deuteroacetylation

The positive ion mode MS spectrum of deuteroacetylated RG-I DP6 confirmed that the sample consists of a derivatized (deuteroacetylated) mixture of monoacetylated RG-I DP6 (m*/z* 1571), diacetylated RG-I DP6 (*m*/*z* = 1568), and triacetylated RG-I DP6 (*m*/*z* 1565) in positive sodiated ion forms [M + Na] ^+^ (Figure 1A). To further investigate the fragmentation behavior, we performed ESI-MS^2^ on the monoacetylated RG-I DP6 (*m*/*z* = 1571). CID fragmentation of the *m*/*z* = 1571 parent ion generated similar daughter products in ESI-MS spectra (Figure 1B; cleavages labeled in blue) as in MALDI-TOF MS^2^ (Supporting Figure S5B). These products included ions at *m*/*z* 1087, 791, 773, 525, and 507, which correspond to Y_4_, C_3_, B_3_, B_3_Y_5_, and B_2_, respectively. The mass of these fragments indicated that the *O*-acetyl group was on one of the three residues closest to the reducing end of the hexasaccharide. Conversely, the B_5_ and Y_2_ cleavages each yielded a set of two ions that were 3 mass units apart (i.e., the difference between an acetyl and a deuteroacetyl group), suggesting the presence of 2 isomers with an *O*-acetyl group in different monosaccharide residues. The B_5_ cleavage gave ions at *m*/*z* 1272 and 1275, and the Y_2_ cleavage gave ions at *m*/*z* 585 and 588. The B_5_ ion with *m*/*z* 1275 was consistent with a fully deuteroacetylated fragment, including the 5 residues from the nonreducing end of Structure E (Figure 1D), and thus proving that the *O*-acetyl group was located on the reducing-end residue; conversely, the B_5_ ion at *m*/*z* 1272 showed the presence of a different parent ion structure (Structure F) that had the acetylation on a sugar other than the reducing-end residue. The Y_2_ ion at *m*/*z* = 588 was consistent with a fully deuteroacetylated fragment, including the two residues closest to the reducing end, and thus demonstrated that the *O*-acetyl group in Structure F was on the second rhamnose residue from the reducing end. Based on the peak intensity ratios of the *m*/*z* = 1275 and *m*/*z* = 1272 B_5_ ions of structures E and F, respectively, the sample has 47% of Structure E and 53% of Structure F.

**Figure 1:**
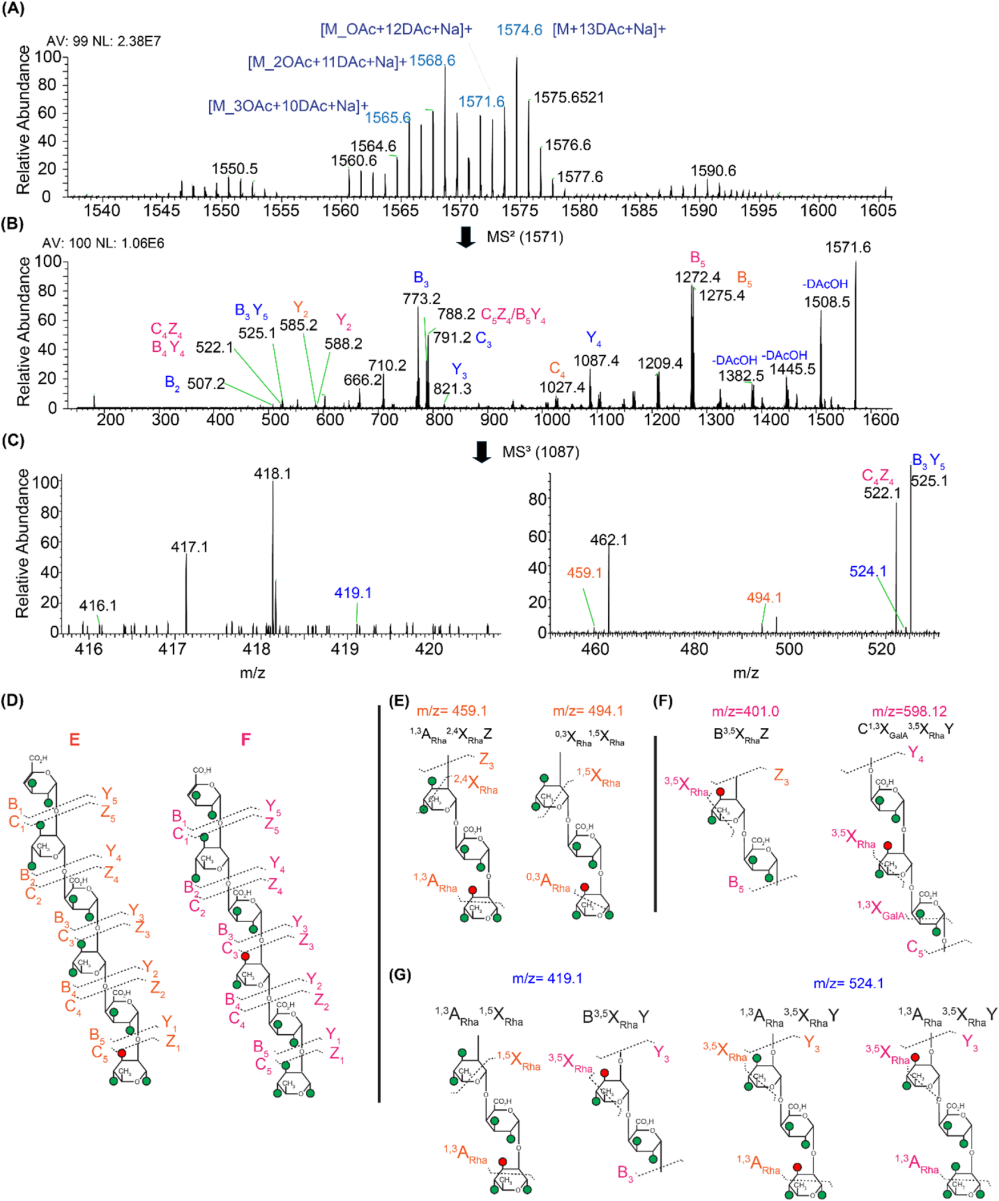
(A) Positive ion mode ESI mass spectra of the deuteroacetylated RG-I DP6. (B) MS^2^ spectrum of the deuteroacetylated monoacetylated RG-I [M_*O*Ac+12DAc+Na]^+^ ion (*m*/*z* = 1571); DAc denotes the deuteroacetyl group. (C) Zoomed in MS^3^ spectrum of *m*/*z* = 1087 to show the diagnostic cross-ring fragments. (D) Two possible acetyl positions were deduced from the MS^n^ spectra Structure E and Structure F. (E) Selected diagnostic cross-ring cleavages of structure E *m*/*z* = 459 and 482. (F) Selected cross-ring fragments for structure F *m*/*z* = 401 and 598. (G) Cross-ring fragments observed for both Structures E and F *m*/*z* 419 and 524. The spectra were obtained in positive mode. The fragments correspond to Structure E labeled in purple, and for Structure F labeled in pink, common fragments for both E and F are labeled in blue. NL: normalized level, AV: averaged number of scans. Nonlabeled peaks are not assigned.

We performed further MS^3^ and MS^4^ experiments to provide additional structural information such as linkages and the position of the *O*-acetyl group in the rhamnose residue (3-*O* or 4-*O*). In the MS^3^ experiment, we selected the ion at *m*/*z* = 1087 (Y_4_) as the precursor ion and subjected it to HCD MS^3^ fragmentation (Figure 1C). This process generated diagnostic cross-ring cleavages that are specifically indicative of the position of the *O*-acetyl group, on either 3-*O* or 4-*O* of the rhamnose residue. The key fragmentation patterns used for this analysis included ^1,3^A or ^0,3^X, or ^3,5^X cross-ring cleavages, where cleavages occurs at the bond between the third (C3) and fourth (C4) carbon atoms of the rhamnose ring (Supporting Figure S7). The *m*/*z* mass list was pulled from the spectra, and each of the theoretical diagnostic cross-ring fragments obtained from GlycoWorkbench was compared with the masses in the spectra to find the diagnostic cross-ring fragments.

The MS^3^ process yielded fragment ions at *m*/*z* = 415, 431, 459, 482, 494, and 477, which correspond to ^1,3^A_Rha_^0,2^X_Rha_Z, ^1,3^A_Rha_^2,5^X_Rha_Z, ^1,3^A_Rha_^2,4^X_Rha_Z_3_, ^3,5^X_Rha_Y_2_, ^0,3^X_Rha_^1,5^X_Rha,_ and ^1,3^A_Rha_^2,4^X_Rha_Y cleavages, respectively (Table 2). As shown in Figure 1E, fragments at *m*/*z* = 459 and 482, the ^1,3^A_Rha_ and ^3,5^X_Rha_ cleavages are consistent with 3-*O*-acetylation on the reducing-end rhamnose, i.e., Structure E (Figure 1E), whereas fragments at *m*/*z* = 401 and *m*/*z* 598 show the cleavages of B_5_^3,5^X_Rha_Z_3,_ and C_5_^1,3^X_Rha_^3,5^X_Rha_Y_4_, which are consistent with acetylation is on the 3-*O* position of the second rhamnose, i.e., Structure F (Figure 1F, Table 2). Additional supporting evidence came from the molecular ion at *m*/*z* = 419 and 524, which gives cleavages for both Structures E and F, showing 3-*O*-acetylation on the rhamnose residue (Figure 1G).

**Table 2:** Summarized Diagnostic Cross-Ring Cleavages for 3-*O*-Acetylation Observed in the Deuteroacetylated Monoacetylated RG-I DP6MS3 Spectra *m/z* = 1087 [M + Na]+ for Structure E and Structure F

diagnostic cross-ring in MS2 (1571) → MS3 (1087)							
for structure E				for structure F			
mass observed	theoretical	mass difference	fragmentation	mass observed	theoretical	mass difference	fragmentation
415.1271	415.1118	0.0152	^1,3^A_Rha_^0,2^X_Rha_Z	401.0339	401.0961	0.0622	B^,3,5^X_Rha_Z
431.1141	431.1067	0.0074	^1,3^A_Rha_^2,5^X_Rha_Z	359.0167	359.0856	0.0689	^0,2^A_GalA_^3,5^X_Rha_Y
459.1397	459.138	0.0017	^1,3^A_Rha_^2,4^X_Rha_Z	598.1209	598.1576	0.0367	C^1,3^X_GalA_^3,5^X_Rha_Y
482.1717	482.1467	0.025	^3,5^X_Rha_Y	627.2243	627.1921	0.0322	^0,2^A_GalA_^0,3^X_Rha_Y
494.1336	494.1467	0.0131	^0,3^X_Rha_^1,5^X_Rha_	669.2688	669.2027	0.0661	B^0,3^X_Rha_Y
477.1486	477.1757	0.0271	^1,3^A_Rha_^2,.14^X_Rha_Y	687.260	687.2132	0.0468	C^0,3^X_Rha_Y
419.1136	419.1067	0.0069	^1,3^A_Rha_^1,5^X_Rha_	419.1136	419.1067	0.0069	B^3,5^X_Rha_Y
524.1692	524.1572	0.012	^1,3^A_Rha_^3,5^X_Rh_Y	524.1692	524.1572	0.012	^1,3^A_Rha_^3,5^X_Rh_Y

This method also showed the possibility of observing two simultaneous cross-ring cleavages from HCD fragmentation. This phenomenon has previously been reported for permethylated oligomers. In these cases, permethylated oligomers yield stable cationic adducts, creating a favorable environment for efficient energy transfer during the collision. This in turn promotes directed fragmentation, increasing the likelihood of observing multiple cross-ring cleavages in a single HCD event.^46^

We also evaluated negative ion mode ESI-MS^n^ on the deuteroacetylated RG-I_*O*Ac DP6 sample for its ability to provide structural information on derivatized RG-I (Supporting Figure S8). The negative ion MS mass spectra showed [M-H]^−^, anionic molecular ion peaks *m*/*z* 1550, 1547, 1544 for nonacetylated, monoacetylated, and diacetylated RG-I DP6. The MS^2^ fragmentation of the *m*/*z* = 1547 monoacetylated RG-I [M-H]^−^ parent ion produced Y_5_, Z_5_, Y_4_, and Y_3_ fragments at *m*/*z* = 1299, 1281, 1045, and 797, respectively. The negative ion mode analysis also shows the two acetyl positions: at the reducing end and on the second rhamnose. These observations are consistent with the expected structure of RG-I and align well with the findings from the positive ion mode analysis (Figure 1). However, MS^3^ of the *m*/*z* = 1045 precursor ion generated MS^3^ spectra (Supporting Figure S8) with low sensitivity and poor signal-to-noise ratio, with ion signals barely distinguishable from background noise, even at reduced HCD collision energies. Therefore, the negative ion mode spectra did not reveal significant information on the acetyl group position. Thus, the negative mode ionization of RG-I ESI-MS^n^ needs further optimization to enhance its effectiveness for the structural analysis of RG-I.

#### Propionylation

Having shown that deuteroacetylation is a reliable method to determine the location of native *O*-acetyl groups in RG-I, we also wanted to test whether propionylation, an alternative derivatization, would provide equal or better results. Propionylation introduces a larger mass shift than deuteroacetylation, which improves the spectral separation and may also enhance the ionization efficiency in mass spectrometry. Additionally, it avoids complications associated with deuterium exchange, potentially resulting in cleaner and more interpretable spectra. For this purpose, we fully pronionylated the partially acetylated RG-I DP6 and analyzed the product using ESI-MS^n^ (Supporting Figure S9). Similarly, this allowed us to determine the *O*-acetyl position in the RG-I oligosaccharides. In the MS^1^ spectrum, ions at *m*/*z* = 1717, 1703, and 1689 (Supporting Figure S9A) corresponded to the fully derivatized nonacetylated, mono-, and diacetylated RG-I DP6, respectively. The MS^2^ analysis of fully propionylated monoacetylated RG-I DP6 (*m*/*z* = 1703) (Supporting Figure S9B) showed that the fragmentation pattern was analogous to that observed in deuteroacetylated spectra (Figure 1), yielding key daughter ions, Y_4_, B_3_, and B_5_Y_4_, respectively. Further fragmenting the ion at *m*/*z* = 1175 (MS^3^, Supporting Figure S9C) led to the identification of diagnostic cross-ring fragments for Structure E at *m*/*z* = 499 and 515 (Supporting Figure S9D), where the acetyl group is on the 3-OH position on the reducing-end rhamnose. The MS^3^ spectra (Supporting Figure S9C) also showed evidence for Structure F by the *m*/*z* = 153 ion, which is characteristic of a B_4_^3,5^X_Rha_Y_3_ cross-ring cleavage on the second rhamnose residue (Supporting Figure S9E).

Both deuteroacetylation and propionylation proved to be effective derivatization methods for determining the *O*-acetyl position in the RG-I oligomers, providing complementary fragmentation patterns that reinforced the structural assignment of the acetyl position. Though it was successful for RG-I DP6, the use of a bulkier acyl group for higher DP oligomers could potentially reduce the solubility due to increased hydrophobicity. Overall, deuteroacetylation is the preferred method, as it offers better resolution for tracking native *O*-acetyl modifications, but both approaches can be used together to enhance confidence in the structural assignment.

### Assessing Migration and the Stability of *O*-Acetyl Group during Derivatization and MS Analysis

The results from ESI-MS analysis of derivatized RG-I oligomers indicated the presence of mono-, di-, and triacetylated species, with *O*-acetylation occurring specifically at the 3-*O* position on the reducing-end rhamnose and inner rhamnose. This agreed with the NMR analysis of the nonderivatized RG-I that showed 3-*O* acetylation in the reducing-end and inner Rha and was consistent with the presence of mono- and diacetylated species. The low-abundance triacetylated RG-I was not detected by NMR, likely due to the lower sensitivity of the method. Importantly, no evidence was observed in the MS data for migration of the *O*-acetyl group in the derivatized samples to the adjacent free hydroxyl group (4-OH) within the same rhamnose residue or to neighboring GalA residues. This confirms that the derivatization conditions and subsequent MS analysis did not induce intramolecular acetyl migration.

Further support for the absence of migration was obtained from ESI-MS analysis of monoacetylated RG-I before and after derivatization (Supporting Figure S10). The fragmentation patterns revealed acetylation either at the reducing end of the rhamnose or at the second rhamnose residue within the chain, resulting in two positional isomers (E and F). The relative ratios of E and F isomers are approximately 1:1 in MS of the derivatized RG-I (Supporting Figure S10). At the same time, the NMR spectrum of the intact sample showed that the degree of acetylation was about the same on the reducing end and the inner rhamnose, which implies that the two monoacetylated species have the same abundance (the diacetylated RG-I does not affect the balance). The unchanged ratios of the monoacetylated isomers before (NMR) and after (MS) derivatization demonstrate that no detectable *O*-acetyl migration occurred across residues under the experimental conditions.

Additionally, no evidence of deacetylation (loss of the *O*-acetyl group) was observed. According to MALDI-MS analysis (Supporting Figure S11), the relative proportion of nonacetylated, monoacetylated, and diacetylated RG-I species remained approximately the same before and after derivatization (Supporting Figure S11), indicating that the *O*-acetyl group was preserved throughout the deuteroacetylation and propionylation procedure.

Taken together, the consistent *O*-acetylation position and stable isomer ratios provide evidence that neither derivatization nor MS analysis induced acetyl migration or loss. These results confirm confidence in the *O*-acetylation patterns assigned under the applied analytical workflow.

### Finding the *O*-Acetyl Position of Celery RG-I Using LC-ESI-MS^n^

To apply our method to an isolated sample with native acetylation, we purified RG-I from celery and digested it with the RG-I lyase enzyme to generate oligosaccharides. We screened the native digested sample using LC-MS, which showed DP4 and DP6 RG-I oligosaccharides with one or two acetyl groups and single galactose branches as components of the sample (Supporting Figure S12). Although the MS^2^ spectra of the underivatized sample confirmed the presence of RG-I oligomers, they did not permit the unambiguous assignment of the acetylated residues. Also, due to the complexity of the mixture and low sample purity, the signals of the target oligomers were difficult to detect, as they were obscured by interfering ions from solvents, buffers, and other coeluting components.

However, these oligosaccharides represent an excellent test case to demonstrate the power of LC-MS^n^ of deuteroacetylated derivatives. Due to the complexity of the background and the diversity in the RG-I oligomers present in the digested celery RG-I sample, the fully deuteroacetylated RG-I oligomers were not detectable by the direct infusion ESI method. Therefore, we analyzed the deuteroacetylated RG-I oligosaccharide sample using nanoflow liquid chromatography (nanoLC), combined with MS^n^ detection (LC-MS^n^) in positive mode, and observed a multitude of sodiated oligomer ions.

The MS profiling of the deuteroacetylated celery RG-I oligomers in LC-MS and MALDI-TOF (Figure 2A and Supporting Figure S13) showed peaks belonging to mono- or diacetylated oligosaccharides derived from four to seven RG-I backbone residues and also showed RG-I oligomers containing a galactose side chain. For each *m*/*z* peak, multiple isomers could be constructed that differed in the placement of the *O*-acetyl group on individual residues and also in the location of the branching in the sequence. This complex mixture of RG-I oligomers showed an LC-MS elution order that correlated with the degree of polymerization, degree of acetylation, the branching pattern, and the length of the branching. The use of a C18 reversed-phase column enabled separation based on hydrophobicity, where oligomers with higher acetylation and longer or more branched side chains exhibited longer retention times due to increased interactions with the column matrix (Supporting Figure S14). The molecular ion of *m*/*z* = 1069 (DP4 RG-I_*O*Ac) eluted first, followed by *m*/*z* = 1366 (DP4 RG-I_*O*Ac with one galactose side chain) and *m*/*z* = 1568 (DP6 RG-I_2*O*Ac). These ions were chosen for further analysis of the acetylation position by tandem MS (Figure 2A).

**Figure 2:**
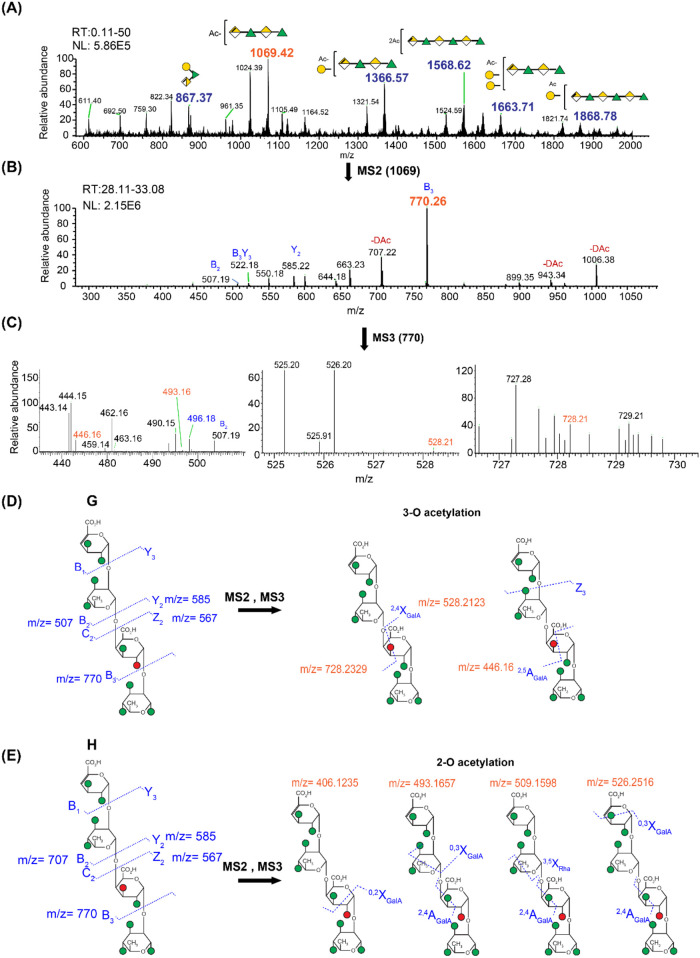
(A) Deuteroacetylated RG-I celery LC-ESI-MS average spectrum in positive ion mode. The spectrum is the average of the retention time (RT), 0.11 to 50 min. (B) Average MS^2^ spectrum (RT 28.11–33.08 min) of the [M + Na] ^+^ ion *m*/*z* = 1069. Symbol nomenclature for glycan (SNFG) shows the identified structures for *m*/*z* in the MS spectra. The ambiguous linkage positions of acetyl and galactose residues are indicated in brackets. (C) MS^3^ spectrum was obtained from the *m*/*z* = 770 fragment ion. The spectrum was the average from 0.3 to 50 min elution, averaged from 1590 scans. The two possible structures identified from the fragments observed in the MS^2^ spectrum of the ion at *m*/*z* = 1069 are (D) Structure G: RG-I 4DP4 with 2-*O*-acetylation in the first GalA residue (from the reducing-end) and (E) Structure H: RG-I 4DP4 with 3-*O*-acetylation in the first GalA residue. Also, the diagnostic cross-ring fragmentation patterns and fragments observed for Structures G and H. LC elution profiles are shown in the Supporting Figure S14.

### *m*/*z* = 1069 – Deuteroacetylated Tetrasaccharide RG-I_OAc Tandem MS Analysis

We selected the perdeuteroacetylated RG-I DP4 backbone with a single *O*-acetyl group (*m*/*z* = 1069) as the precursor ion and analyzed it by MS^2^ using CID. The fragment ions obtained [*m*/*z* = 770 (B_3_), 585 (Y_2_), 507 (B_2_)] indicated that the *O*-acetyl group was primarily located on the GalA residue (Figure 2B) rather than on the reducing-end rhamnose [*m*/*z* = 773 (B_3_)], which accounted for less than 2% of the total population (Supporting Figure S15).

Further MS^3^ analysis of the *m*/*z* = 770 (B_3_) fragment ion using HCD generated both glycosidic and cross-ring fragments (Figure 2C). Comparison of the observed fragment ions with theoretical fragments generated using GlycoWorkbench identified diagnostic cross-ring ions at *m*/*z* = 446, 528, 728, and 409, corresponding to ^2,5^A_GalA_Z, ^2,4^X_GalA_, ^0,2^A_GalA,_ and ^0,2^X_GalA_ fragments (Supporting Table S3). These fragments confirmed that the major site of *O*-acetylation was at the 3-*O* position of the GalA residue (Figure 2D). In addition, minor diagnostic fragments consistent with 2-*O*-acetylation were also detected, indicating a small proportion (∼14%) of 2-*O*-acetylated GalA (Figure 2E). Overall, the RG-I DP4 backbone was predominantly 3-*O*-acetylated on the GalA residue (approximately 86%) (Supporting Figure S16).

### *m*/*z* = 1366 Deuteroacetylated Branched Pentasaccharide RG-I_OAc Tandem MS Analysis

The addition of branching significantly increases the complexity of the *O*-acetylated RG-I. The molecular ion *m*/*z* = 1366, which comes from an RG-I pentasaccharide comprising a DP4 RG-I backbone with a single galactose branch and one acetyl group. The Gal residue can be either on the reducing-end Rha (Structures J, K, and L in Figure 3C and Supporting Figure S17) or the rhamnose next to the nonreducing end (Structures M, N, and O in Supporting Figure S17).^38^ Together with the various possible positions of the *O*-acetyl group, this gives rise to multiple isomers.

**Figure 3:**
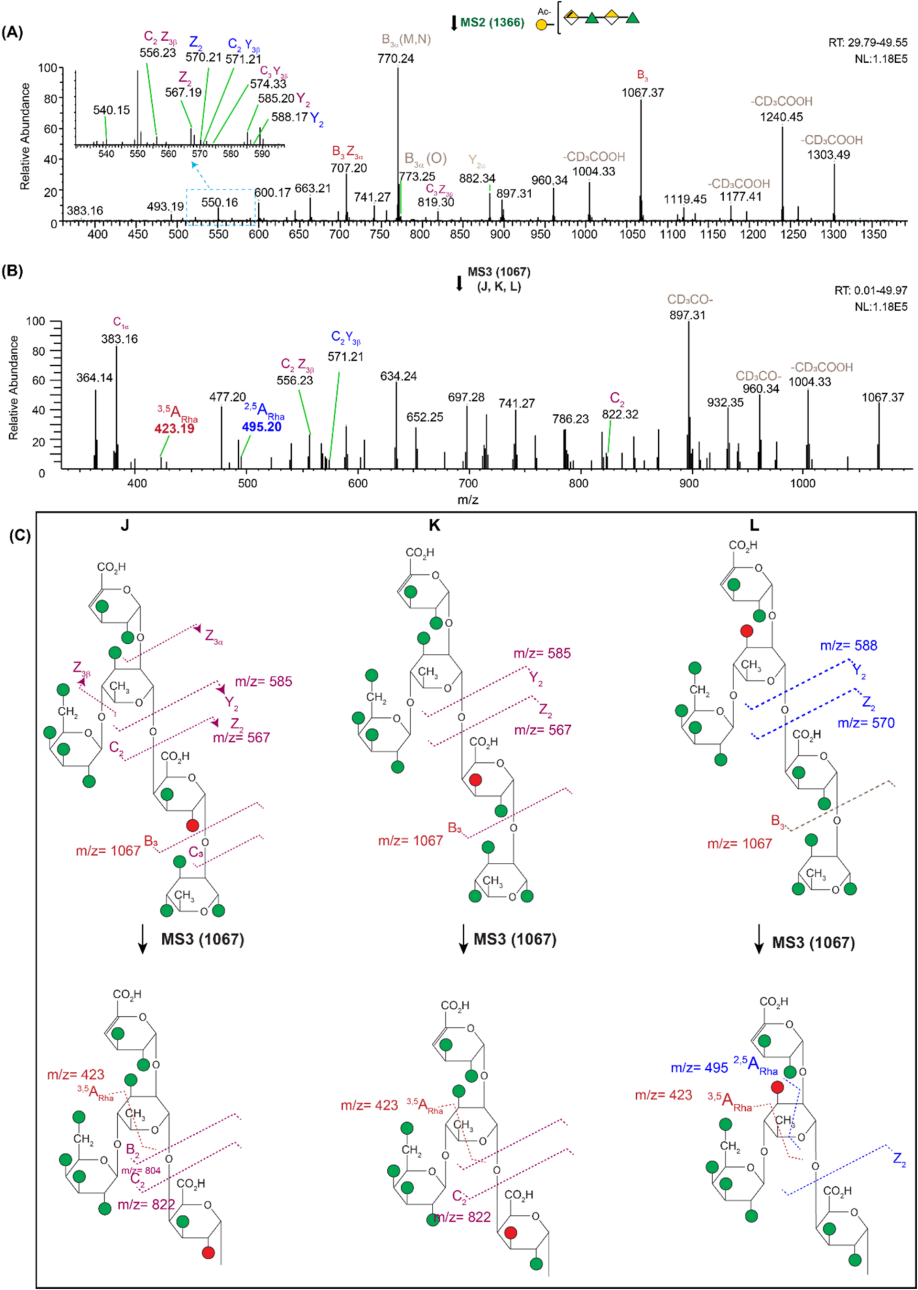
(A) MS^2^ spectrum of *m*/*z* = 1366 [M + Na]+; the inset shows the zoom- in region marked in blue box. The spectrum is averaged from RT 29.79–49.55 min. (B) MS^3^ spectrum of *m*/*z* = 1067 for J, K, and L isomers averaged from RT 0.01 49.97 min. (C) J, K, and L structures and cleavages observed in the MS spectra are labeled. The red circle denotes the *O*-acetyl groups, and the green circles are deuteroacetylated groups. The *m*/*z* 423 diagnostic cross-ring cleavage present in J, K, and L confirms that the rhamnose is branched at the 4-*O* position. NL: normalized intensity level. The structures of the nonlabeled peaks are not determined.

To narrow down the possible isomers and to get more structural information, we isolated the molecular ion at *m*/*z* = 1366 and subjected it to MS^2^ analysis (Figure 3A). The resulting spectrum revealed fragment ions that provided critical insight. The fragment at *m*/*z* = 1067 (B_3_ cleavage) was consistent with the structure of J, K, and L. Conversely, the fragment at *m*/*z* = 770 (B_3α_) was only consistent with the M and N isomers, indicating 2-*O* or 3-*O*-acetylation on the GalA residue (Supporting Table S3 and Figure S18C). Further diagnostic ions at *m*/*z* = 585, 556, 567, and 574 (Y_2_, C_2_Z_3β_, Z_2_, and C_2_Y_3β_) were observed exclusively in the J and K isomers (Figure 3C and Supporting Table S4). Ions at *m*/*z* = 588, 571, and 570 (Y_2_, C_3_Y_3β,_ and Z_2_) were specific only to the L isomer (Figure 3C**:** Structure L), and *m*/*z* 773 and 616 were for the O isomer, suggesting the presence of the 3-*O*-acetylation on the branching rhamnose (Supporting Table S3). The MS^2^ spectrum showed fragments indicative of *O*-acetylation on both the GalA and Rha residues. These isomers are coeluting together and cannot be separated chromatographically (Supporting Figure S19).

To gain a deeper understanding of the specific isomers present (J, K, and L isomers), we performed MS^3^ analysis on the *m*/*z* = 1067 B_3_ fragments (J, K, and L Figure 3B and Supporting Table S5). The MS^3^ spectrum confirmed the presence of *O*-acetyl groups on both GalA and Rha, further supporting the identification of the J, K, and L isomers. A fragment at *m*/*z* = 423.19 confirmed the galactose branching on the 4-*O* position of rhamnose, corresponding to a ^3,5^A_Rha_ cleavage for J, K, and L isomers (Figure 3C and Supporting Table S5). These results support the conclusion that the second rhamnose residue is linked as → 2,4-α-L-Rha-1→, leaving only the 3-OH position free.

The branching galactose at the 4-*O* position having been established, the fragments at *m*/*z* = 495, 507, 668, 717, and 745 (corresponding to ^2,5^A_Rha_, ^1,5^A_Rha_Z, ^3,5^A_GalA_Z, B^1,3^X_Rha_, and ^1,4^A_Rha_, respectively, see Figure 3C) prove that the rhamnose acetylation is present on the 3-*O* position, confirming Structure L. On the other hand, it is challenging to definitively assign 2-*O*/3-*O*-acetylation in the GalA residues (J and K) with the current MS data due to many possibilities of cleavage pathways and multiple coexisting isomers and fragments.

To determine the location of the *O*-acetyl group on the GalA residue, we performed MS^3^ of *m*/*z* 770 (obtained from 1366 MS^2^) (Supporting Figure S18). The spectra revealed diagnostic peaks for both 3-*O*-acetylation (M isomer) and 2-*O*-acetylation (N isomer) (Supporting Figure S18B,C and Table S5).

#### *m*/*z* = 1568 – Perdeuteroacetylated Linear Hexasaccharide RG-I_2OAc Tandem MS Analysis

We also studied perdeuteroacetylated DP6 RG-I with two acetyl groups (*m*/*z* = 1568) with tandem MS (Supporting Figure S20). The MS^2^ CID spectrum of the *m*/*z* = 1568 gave daughter ions characteristic of P, Q, R, S, and T (Supporting Figure S20a,b). The product ion *m*/*z* = 1084 (Y_4_), ion confirms the P, Q, R, S, and T, and *m*/*z* = 785, 1269 confirms the P and Q structures (Supporting Figure S20). B_3_ (*m*/*z* = 770) showed that two acetyl groups are like in the structure Q, R, S, and T. Further MS^3^ analysis of *m*/*z* = 1084 (Supporting Figure S20C) gave diagnostic cross-ring peak *m*/*z* = 461.15 cleavage ^2,5^A_GalA_Y_3_, which shows the presence of 3-*O*-acetylation in the GalA residue adjacent to an acetylated rhamnose corresponding to Structure P (Supporting Figure S20D; Structure P). However, due to the complexity of the structure (many possible *O*-acetylation positions) and due to limitations in the diagnostic cross-ring cleavages, the *O*-acetylation position of the rhamnose (P isomer) or other structures cannot be confirmed.

Overall, the current structural analysis of perdeuteroacetylated celery RG-I oligomers using the LC-ESI-MS^n^ revealed a highly heterogeneous composition with multiple isomers arising from variations in acetylation and branching, highlighting the structural complexity of celery RG-I that was not studied before.

To complement the MS data, we performed an NMR analysis on the native RG-I from celery (undigested). The NMR analysis provided unambiguous evidence for rhamnose 3-*O*-acetylation and the presence of branching galactose on the acetylated rhamnose residues in RG-I as described previously (Supporting Figure S21).^38^ In addition, the NMR spectra showed the presence of prominent signals of acetyl groups that were likely in part due to GalA acetylation in homogalacturonan, complicating the detection of signals of RG-I GalA acetylation that could be relatively minor.^11,38^ Due to the heterogeneity of GalA acetylation, leading to signal broadening and overlaps, we did not attempt to analyze RG-I GalA acetylation by NMR in the intact celery RG-I sample. The combination of depolymerization, derivatization, and ESI-MS^n^ of the oligomers offers a faster and more reliable approach for identifying *O*-acetylation sites, and it also provides the capability of seeing minor modifications (such as acetylation of GalA in RG-I), which cannot be observed by NMR. While further purification of digested RG-I oligomers could facilitate *O*-acetyl position determination using NMR, its applicability is constrained by the relatively high sample quantity that is required. Thus, MS proves to be a more effective method for pinpointing *O*-acetylation sites compared to NMR in the case of intact RG-I.

## Conclusion

In conclusion, this study demonstrates the effectiveness of derivatization combined with advanced tandem mass spectrometry approaches, including CID and HCD for the structural elucidation of RG-I oligomers with distinct *O*-acetylation patterns. By employing combinatorial fragmentation strategies and leveraging the LC-MS separation technique, we enhanced the resolution of complex mixtures, facilitating precise *O*-acetyl group localization. The introduction of trideuteroacetyl and propionyl modifications successfully stabilized *O*-acetyl groups, mitigating challenges associated with acetyl migration and loss during MS^n^ analysis, and increased the sensitivity to pinpoint the *O*-acetyl position. Derivatization also improved sensitivity by virtue of the enrichment of the more hydrophobic derivatized carbohydrates by extraction from water-soluble components. However, conventional CID and HCD methods primarily generate glycosidic and limited cross-ring cleavages, sometimes limiting the ability to differentiate between closely related isomers when it comes to complex mixtures. Incorporating ultraviolet photodissociation (UVPD),^47^ electronic excitation dissociation (EED),^48^ electron transfer dissociation (ETD), and electro impact excitation of ions from organics (EIEIO)^49^ could overcome these limitations by providing enhanced diagnostic cross-ring fragmentation pathways, preserving labile modifications, and offering more comprehensive structural characterization. This approach provides valuable insights into pectin *O*-acetylation with implications for understanding the functional roles of RG-I modifications in plant biology. Furthermore, this strategy can be extended to other acylated oligosaccharides, offering a powerful tool for studying enzymatic regiospecificity and structural diversity in complex polysaccharides.

## Supplementary Material



## Data Availability

All relevant data that support the findings of this study are available within the article and Supporting Information.

## Notes

MALDI-TOF spectra of [M + Na]+ propionylated DP6, DP8, and DP10 RG-I. Pro denotes propionyl groups; derivatization of partially acetylated RG-I (RG-I_OAc DP6); NMR acquisition parameters; glycosidic cleavage fragments observed in the MS2 spectra of m/z 1366 characteristics for different isomers (PDF)
