# Supplementary material for: Mass Spectrometric Determination of Site-Specific O‑Acetylation in Rhamnogalacturonan I Oligomers
Source: J Am Soc Mass Spectrom. 2026 Mar 30;37(5):1160–72. doi: 10.1021/jasms.5c00440 (PMC13154352; doi:10.1021/jasms.5c00440)
Supplement: Supplementary file 1 [file js5c00440_si_001.pdf]

Supplementary Information

Mass Spectrometric Determination of Site-Specific *O*-Acetylation in Rhamnogalacturonan I Oligomers

Liyanage Devthilini Fernando [a], Xu Yang [a], Stephanie Archer-Hartmann [a], Lubana Shahin[a], Liang Zhang [a], Breeanna R. Urbanowicz [a], Christian Heiss [a], and Parastoo Azadi [a]*

[a] Complex Carbohydrate Research Center, University of Georgia, Athens, GA 30602, USA

*Correspondence: azadi@ccrc.uga.edu

Supplementary Methods 1:

Summarizing the General method for Determining RG-I Acetylation Sites by tandem mass spectrometry

1. Sample Preparation:
   The partially *O*-acetylated oligosaccharides or polysaccharide fragments are isolated and purified, (partial hydrolysis or enzymatic digestion to produce oligomers). Derivatize the oligomers (deuteroacetylation or propionylation).

2. Mass spectroscopy ionization:
   The sample is introduced into the mass spectrometer using ESI (electrospray ionization) or LC-MS generating molecular ions (either positive or negative ion mode).

3. Selection of precursor ion: A specific *O*-acetylated molecular ion (e.g., $[M–H]^-$/[M-Na]+) corresponding to the desired degree of polymerization (DP) and acetylation is selected for fragmentation.

4. Fragmentation (MS^2/MS^n analysis): The precursor ion is subjected to collision-induced dissociation (CID) or higher-energy collisional dissociation (HCD).

5. Interpretation of fragment ions:

Glycosidic cleavages (B/Y or C/Z ions) indicate the sequence of monosaccharide residues.

Cross-ring cleavages (A- and X-type ions) provide structural information about substitutions (e.g., acetylation sites) on specific sugar residues.

- Predict the possible structures in GlycoWorkbench
- Narrow down the predicted structures by comparing the MS^2 daughter ion obtained.
- Predict the possible diagnostic cross-rings based on the *O*-acetyl position on the residues using GlycoWorkbench
- The *m/z* mass list is pulled from the spectra and each of the theoretical diagnostic cross-ring fragments obtained from GlycoWorkbench was compared with the masses in the spectra to find out the diagnostic cross-ring fragments.

6. Validation and cross-verification:
   The proposed acetylation sites are confirmed by comparing experimental spectra with theoretical fragmentation patterns, or complementary techniques such as NMR spectroscopy.

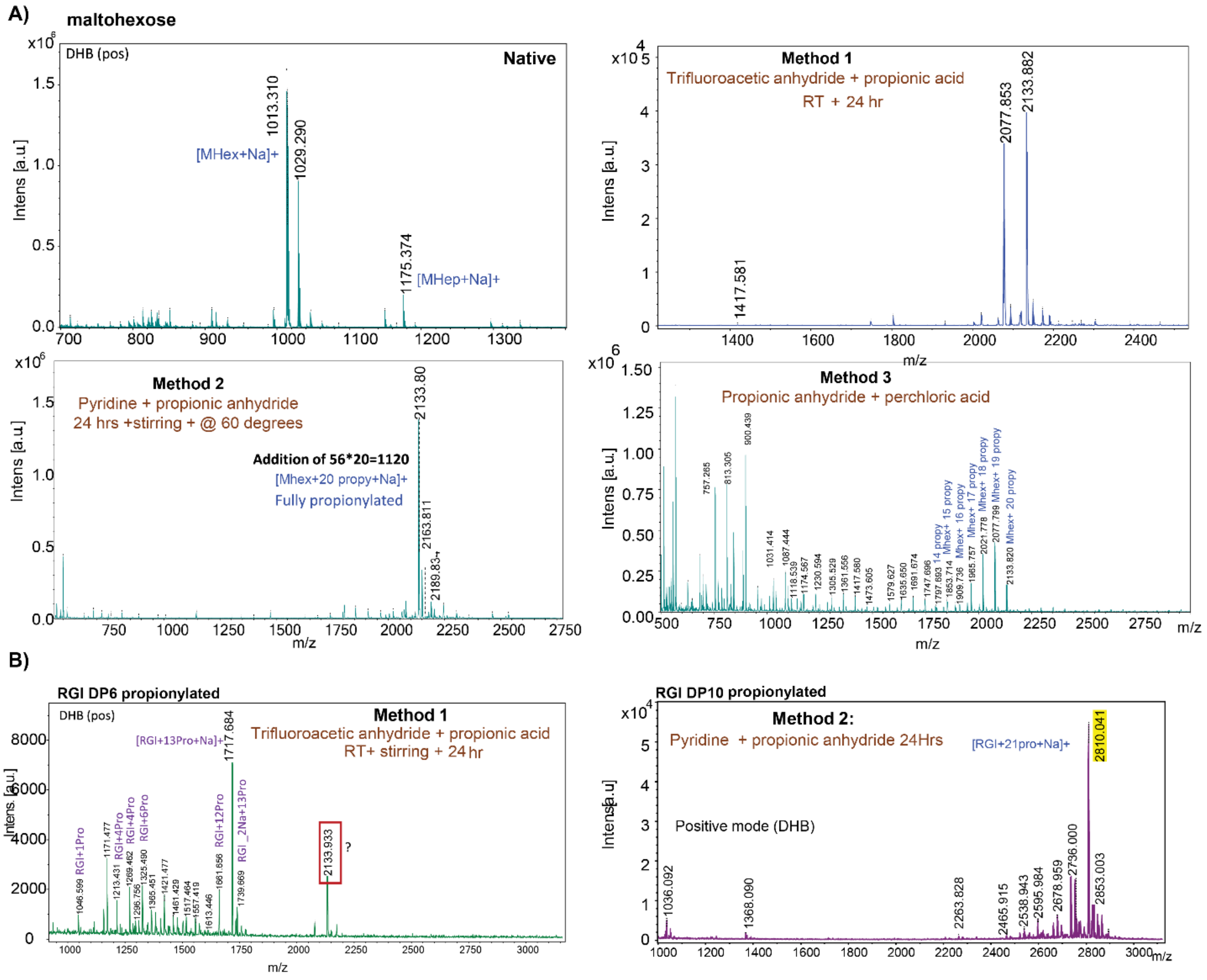

Supplementary Figure 1: MALDI-TOF spectra of **A**) Maltohexaose (underivatized) and acylated maltohexaose with three different methods; Method **1**- trifluoracetic anhydride + propionic acid, Method **2**- pyridine + propionic anhydride, Method **3**- Propionic anhydride +perchloric acid. **B)** Derivatized RG-I DP6 with Method **1** (left) and Method **2** (right).

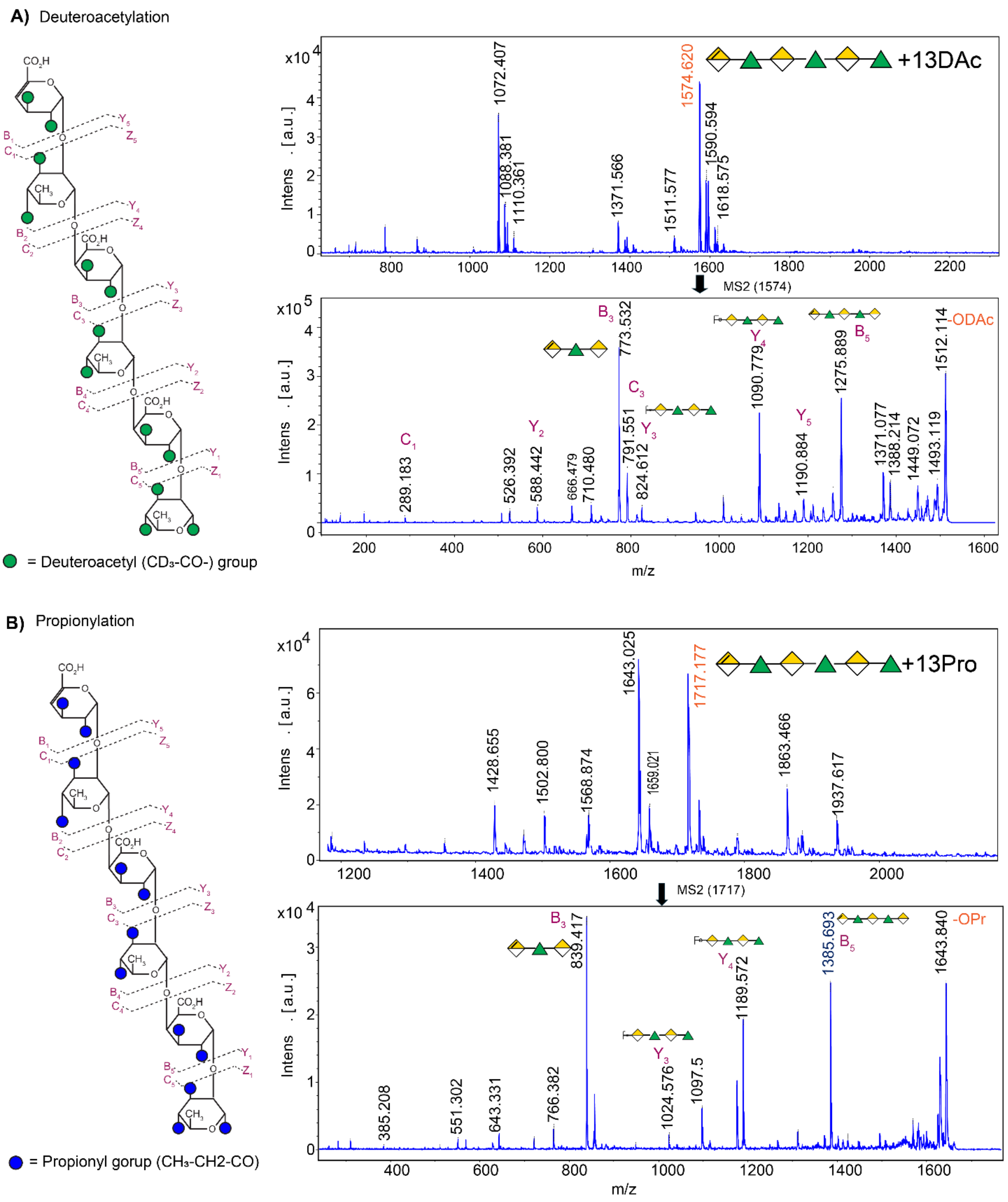

Supplementary Figure 2: MALDI-TOF spectra of [M+Na]+ **A)** Deuteroacetylated RG-I DP6 (m/z 1574 represent fully deuteroacetylated RG-I and the MS^2 spectra of the *m/z* = 1574 parent ion. **B)** Propionylated RG-I DP6 and the MS^2 spectra of the *m/z* = 1717 parent ion. -DAc denotes deter acetyl groups and "-Pro" denotes propionyl groups.

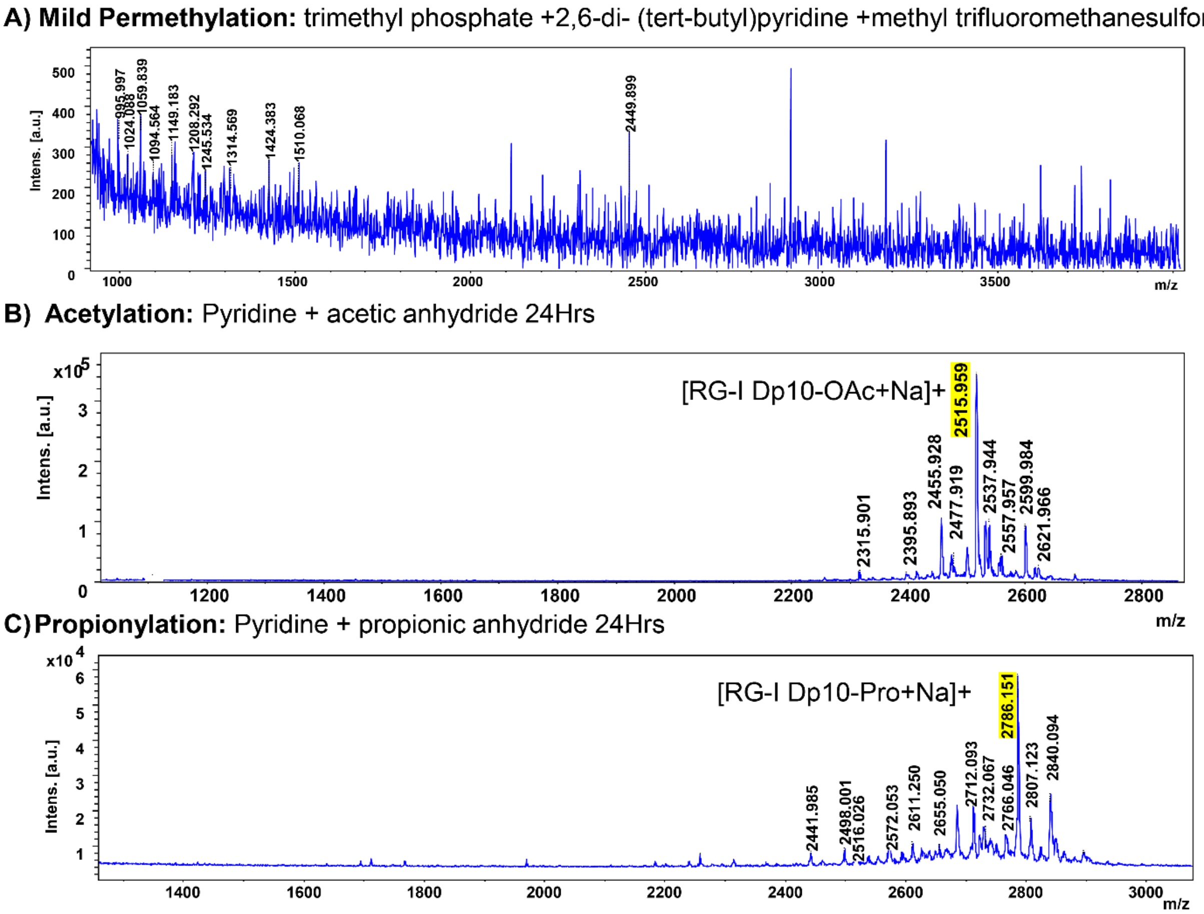

Supplementary Figure 3: MALDI-TOF spectra of [M+Na]+ **A)** mild permethylated RG-I DP6 **B)** acetylated RG-I DP 10 and C) Propionylated RG-I DP10.

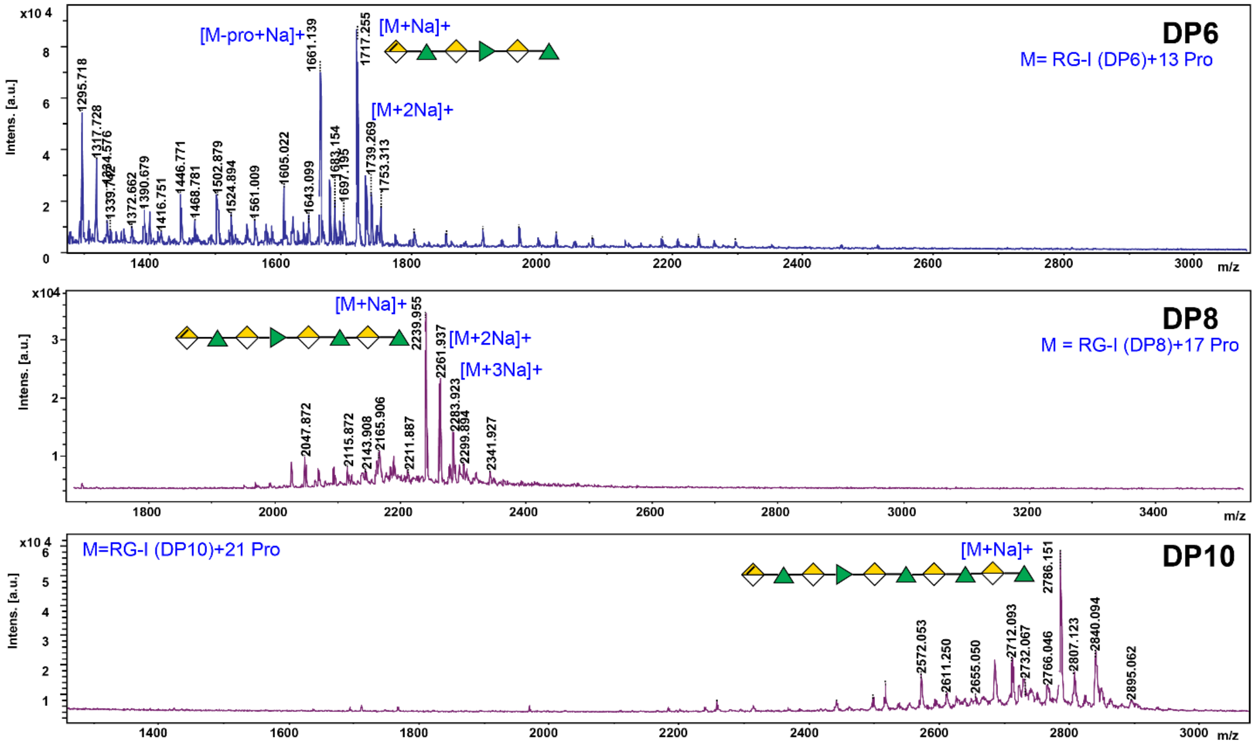

Supplementary Figure 4: MALDI-TOF spectra of [M+Na]+ propionylated DP6, DP8 and DP10 RG-I. Pro denotes propionyl groups.

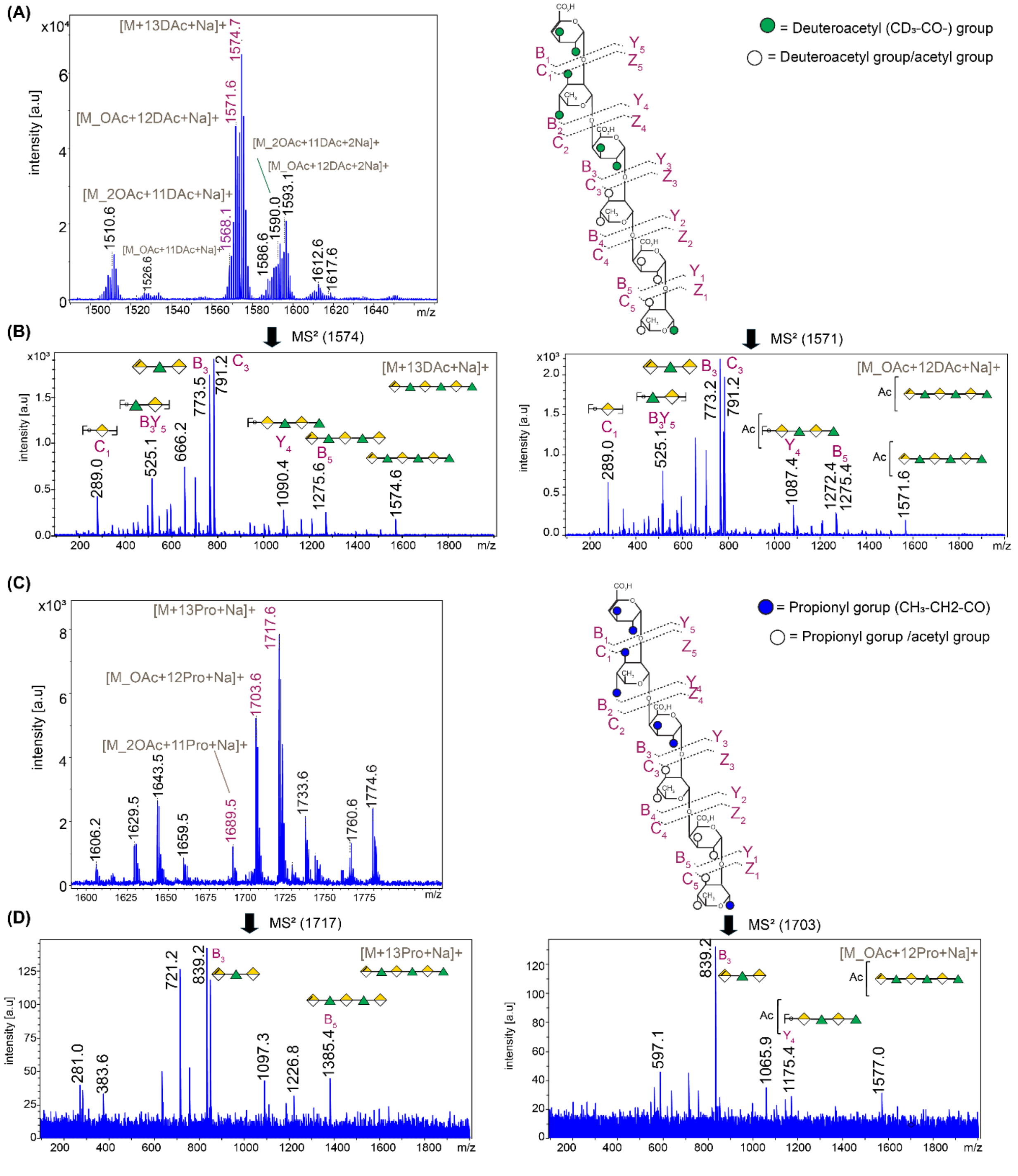

Supplementary Figure 5: Derivatization of partially acetylated RG-I (RG-I_*O*Ac DP6). **(A)** Deuteroacetylated RG-I MS[1] spectrum (left), RG-I structure, green circles denote deuteroacetyl groups white circles denote either deuteroacetyl or acetyl groups. **(B)** MS[2] spectra for *m/z*=1571 [RG-I_*O*Ac DP6+Na]$^+$ and *m/z* =1574 precursor ion corresponds to [RG-I DP6+Na]$^+$. **(C)** Propionylated RG-I DP6 MS spectrum (left), RG-I structure, green circles denote deuteroacetyl groups white circle denotes either deuteroacetyl or *O*-acetyl groups (right). **(D)** MS[2] spectra of *m/z* =1717 [RG-I DP6+Na]$^+$ and *m/z*=1703 [RG-I_DP6*O*Ac+Na]$^+$. Fragmentation nomenclature shown according to Domon and Costello[1] for RG-I. Based on the MALDI-TOF spectra the whites circles shows the possible positions of acetyl groups. The non-labelled peaks, the structural composition is not deducted. Both deuteroacetylated and propionylated RG-I showed a mixture of non-acetylated, mono-acetylated and diacetylated RG-I DP6 in the sample.

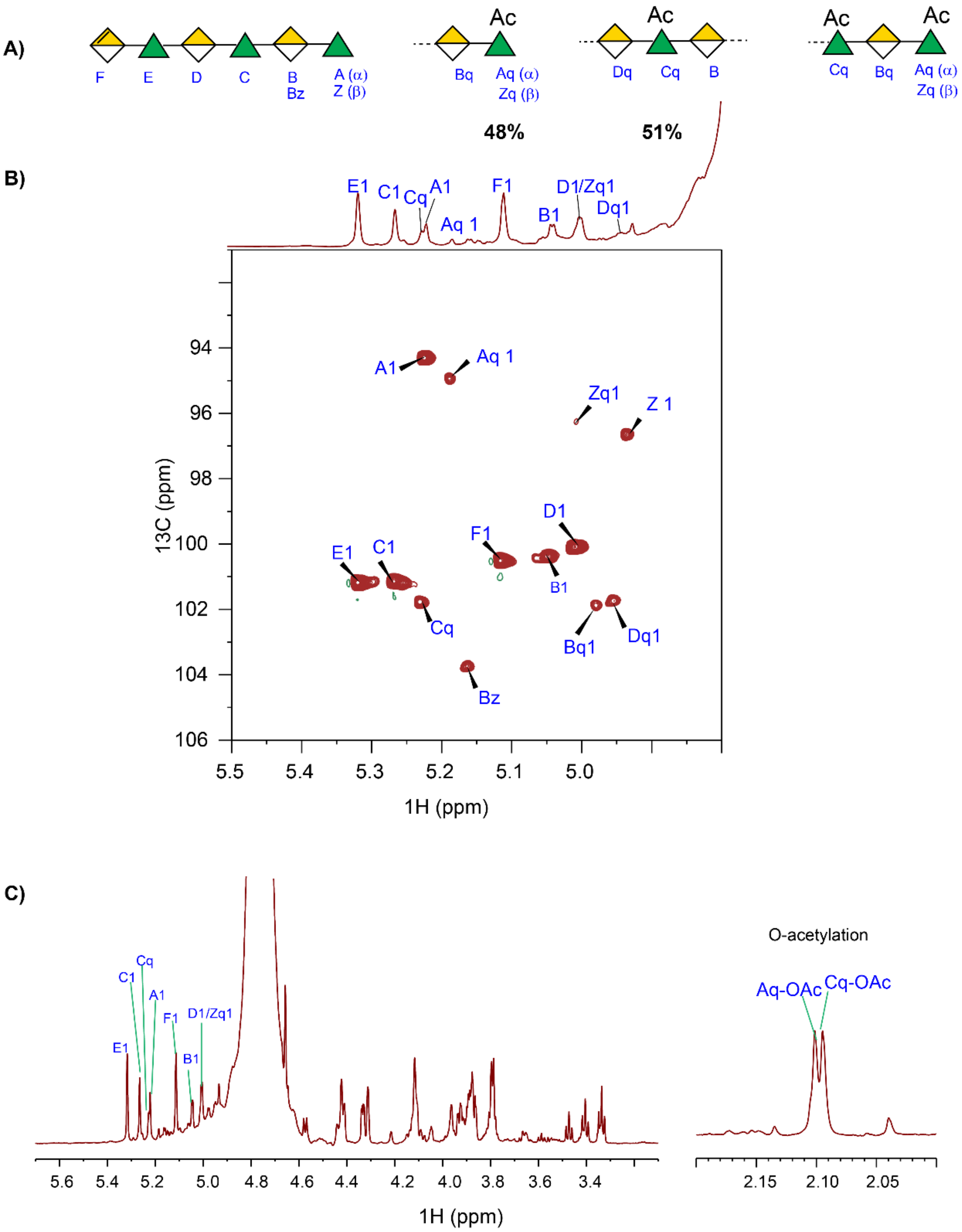

Supplementary Figure 6: 1D ^{1}H and 2D ^{1}H-^{13}C HSQC spectra of the partially acetylated RG-I DP6 (non-derivatized). **A)** Structures of the non-acetylated and partially acetylated RG-I DP6 are present in the sample. **B)** The 2D HSQC spectrum shows the diagnostic anomeric peaks for the non-acetylated RG-I DP6 and two mono-acetylated RG-I DP6. The HSQC shows 3-*O*-acetylation in the reducing-end rhamnose and 3-*O*-acetylation in the second rhamnose**. C)** The 1D NMR of the partially acetylated RG-I DP6. The acetyl group signals are shown on the right (scales are not identical with left). The signals at 2.09 and 2.10 ppm are due to the *O*-acetylation of the internal and reducing-end rhamnose residues. The assignments could be interchangeable, but the about-equal intensities of the separate acetyl signals show that the relative ratio of acetylation at the reducing end and in the second rhamnose is ~1:1. The 3-*O*-acetylation ratios for reducing end Rha (Aq and Zq) and the second rhamnose (Cq) were also obtain from the 2D HSQC spectra also confirms ~1:1 ratio.

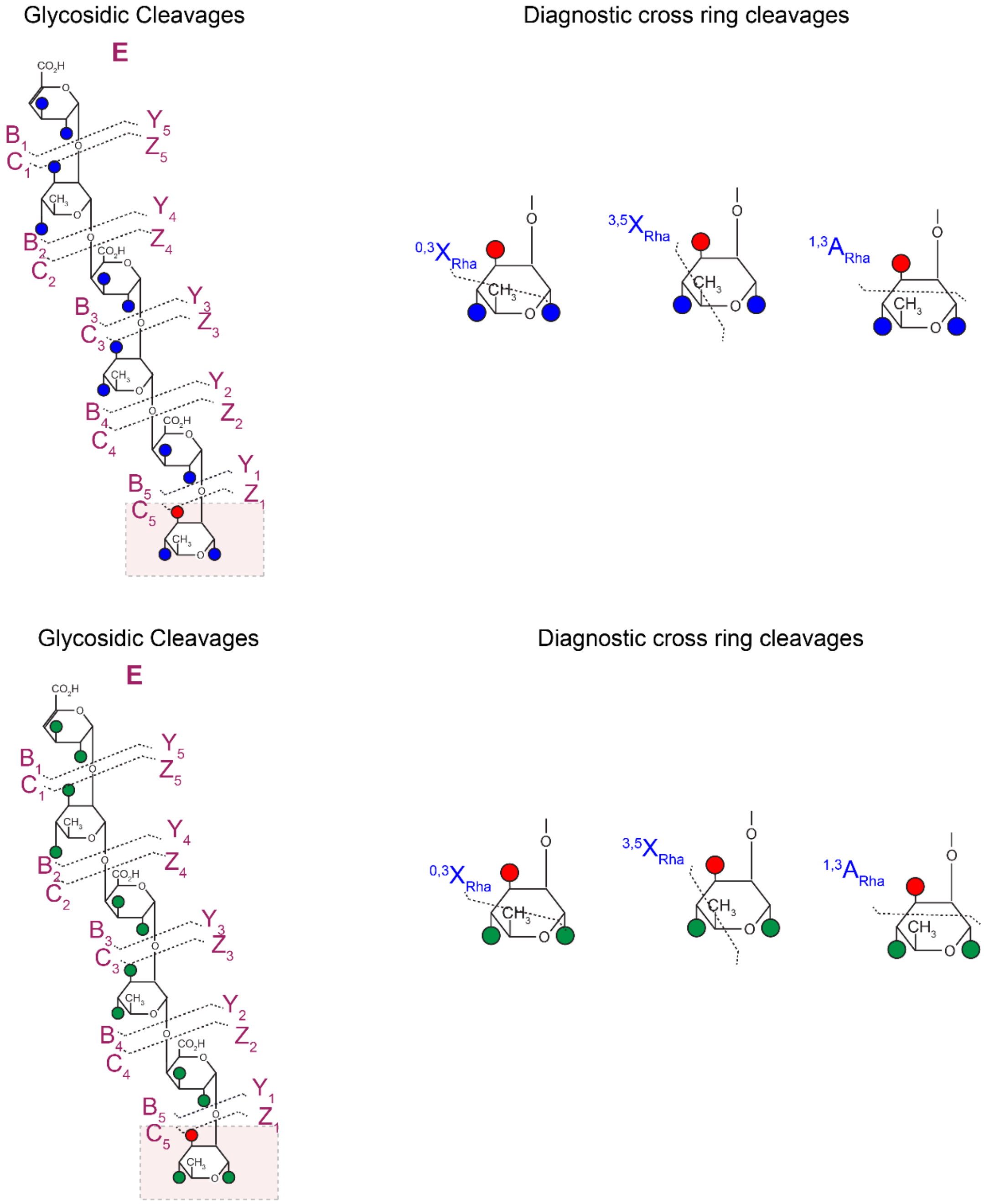

Supplementary Figure 7: The diagnostic cross-ring cleavages (A and X cleavages) for DP6 RG-I_*O*Ac. The Fragmentation nomenclature according to Domon and Costello. In order find the position of the acetyl group the cross-ring cleavage needs to happen in the covalent bond between two carbons connected to the acetyl group and the free hydroxyl group. Blue circle denotes the free hydroxyl with derivatization with propionyl groups and green circle denotes the deuteroacetylation, red circle: acetyl group.

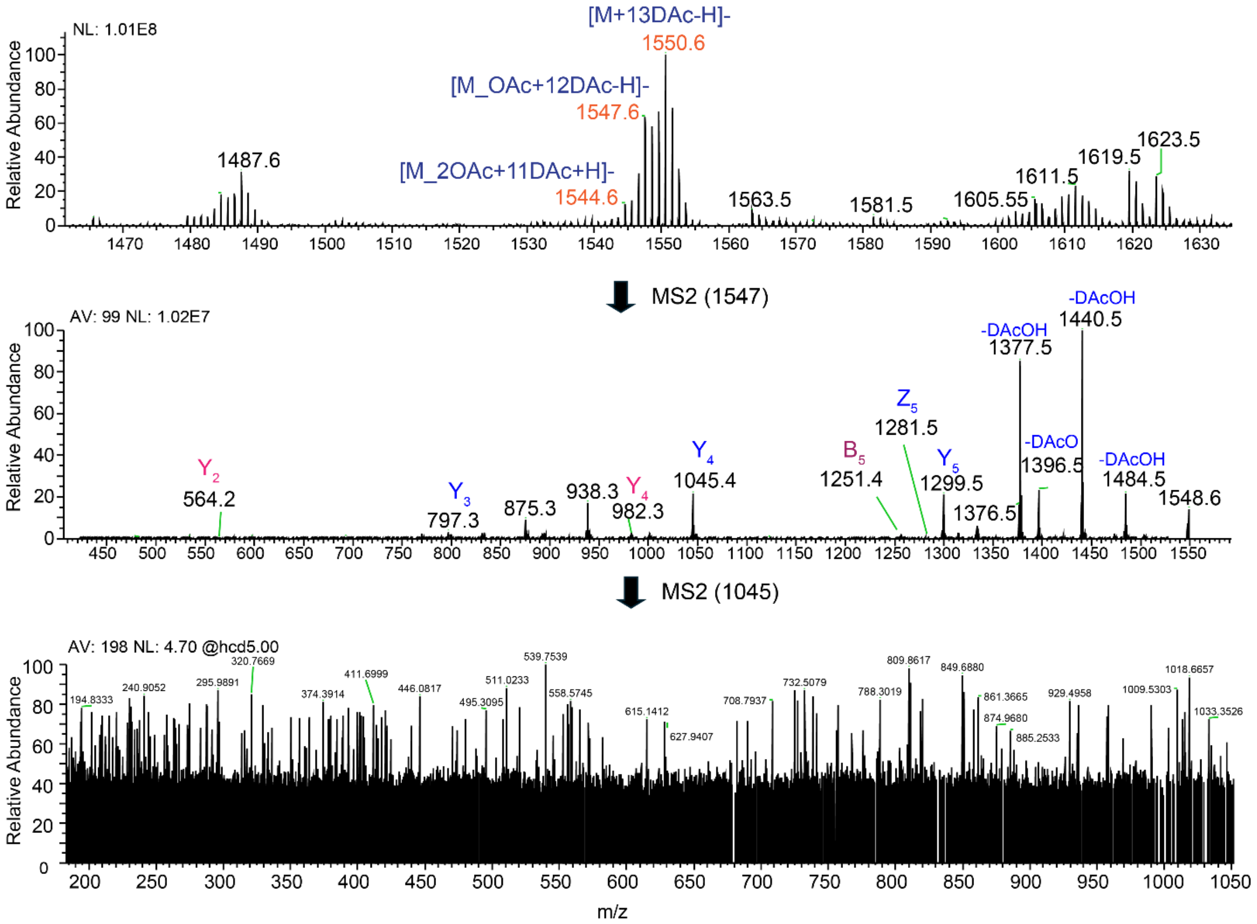

Supplementary Figure 8: ESI-MS^n spectrum of perdeuteroacetylated, monoacetylated RG-I DP6 (top) in negative ion mode. The MS^2 spectrum of m/z 1547 $[M-H]^-$ parent ion (middle). The MS^3 was obtained from the m/z= 1045 molecular ion (bottom). NL: normalized level, AV: averaged number of scans. Non-labeled peaks composition is not determined.

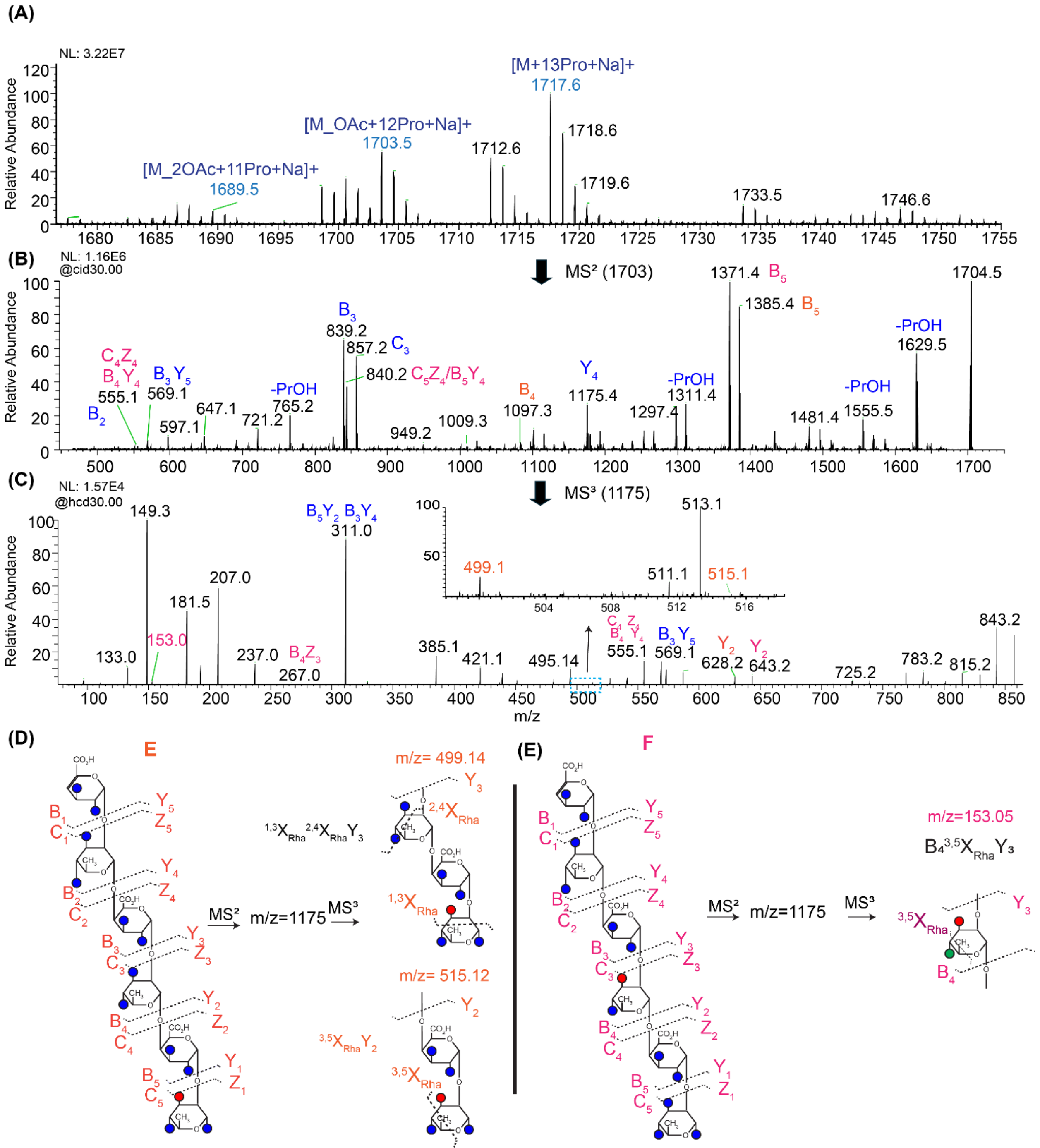

Supplementary Figure 9: **(A)** ESI mass spectra of the propionylated RG-I DP6 in positive ion mode. **(B)** MS^2 spectrum of the [M_*O*Ac+12Pro+Na]$^+$ ion *m/z*=1703, Pro denotes propionyl groups. **(C)** MS^3 spectrum of *m/z* = 1175. The inset shows the zoom in region mark in blue box. **(D)** Fragmentation pattern and the diagnostic cross ring fragment observed for structure E, where acetylation is on the 3-*O* position of the reducing-end rhamnose. **(E)** Diagnostic cross-ring cleavage for structure F *m/z* = 153, where acetylation on the 3-*O* position of the second rhamnose. The blue circle on the structures denotes propionyl groups and red circle shows the acetyl position. The fragments correspond to Structure E labeled in orange, and for Structure F labeled in pink, common fragments for both E and F are labeled in blue. NL: Normally, the structures of the non-labeled peaks could not be determined.

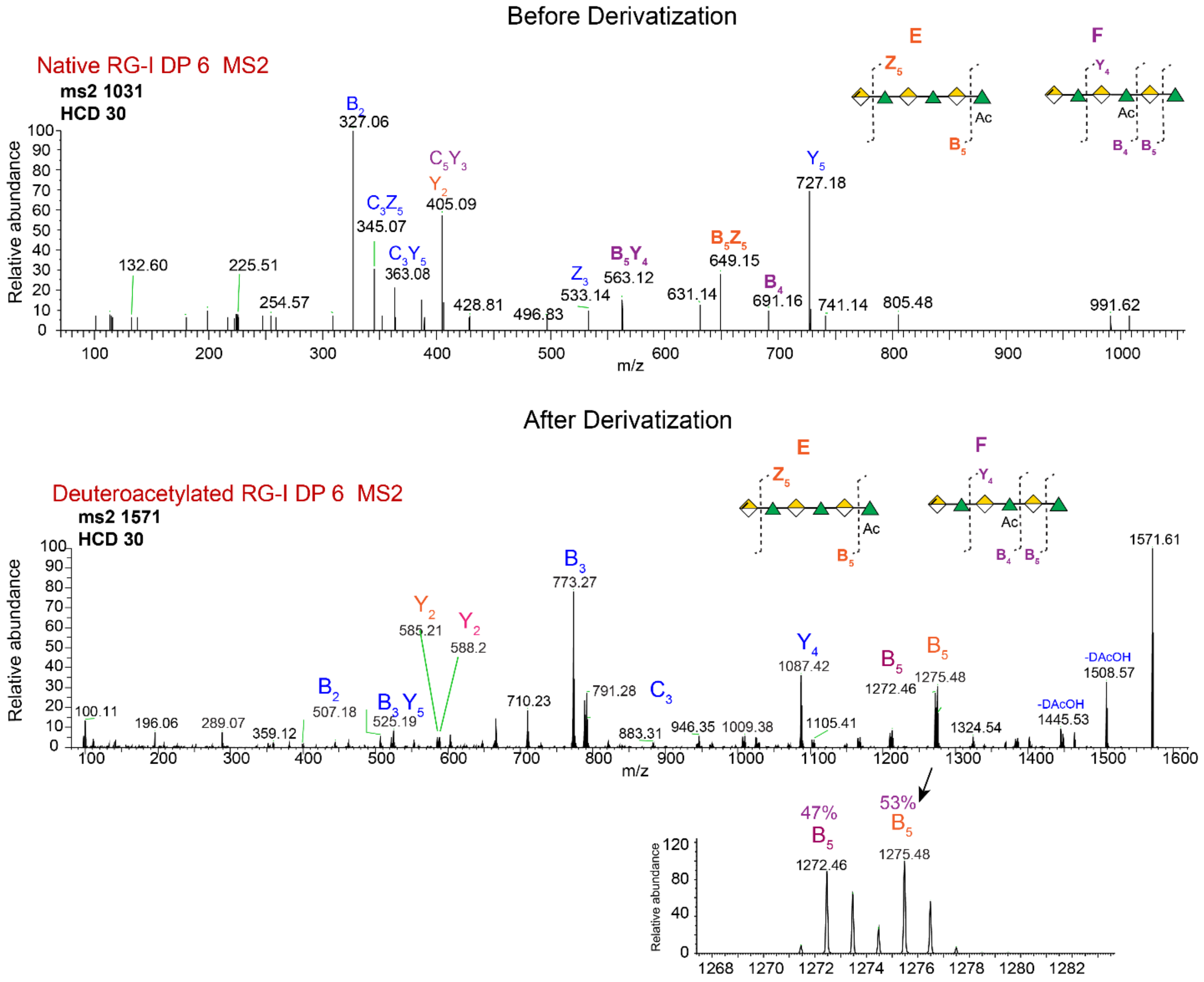

Supplementary Figure 10: ESI-MS^2 analysis of before and after derivatization of the mono acetylated RG-I DP6. MS^2 of 1031 of mono acetylated RG-I DP6 (top). MS^2 of perduteroacetylated RG-I of m/z =1571 (bottom) the fragmentation patterns show the presence of acetylation on reducing end rhamnose (E) and the second rhamnose (F). The zoomed-in region in deuteroacetylation spectra shows the E and F isomers are present in approximately in 1:1 ratio.

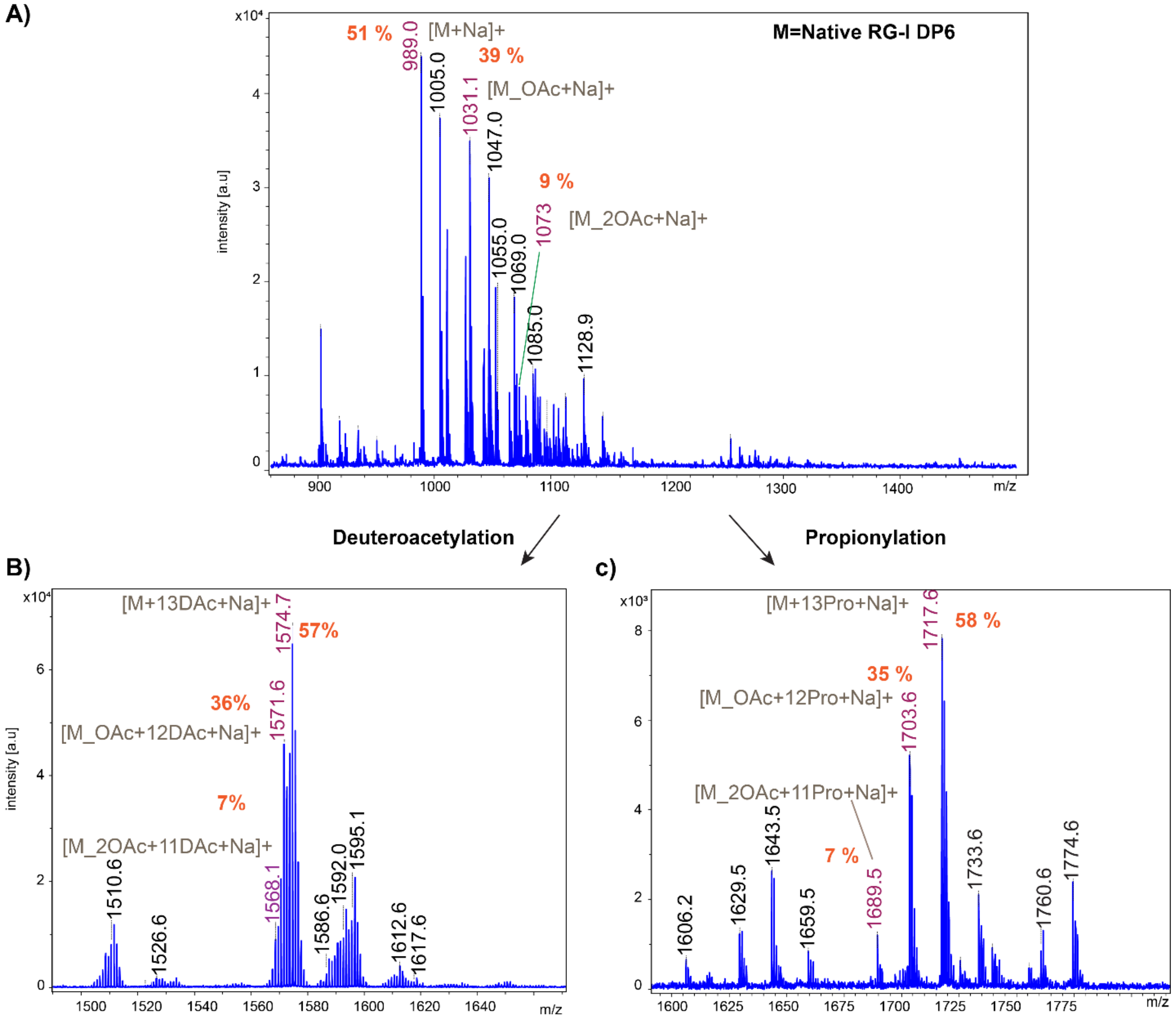

Supplementary Figure 11: Enzymatically mono acetylated RG-I DP 6 oligomer MALDI-TOF MS spectra in positive ion mode $[M+Na]^{+}$. The ratio between non-acetylated, mono acetylated and di acetylated RG-I DP6 remain relatively same after derivatization **A)** MALDI-TOF MS spectra of the native underivatized oligomer shows a mixture of non-acetylated RG-I (m/z =989; 51%) and mono acetylated RG-I DP6 (m/z=1031;39%), minor amount of diacetylated RG-I DP6 (m/z=1073; 9%) **B)** Fully deuteroacetylated RG-I DP6 MALDI-TOF MS spectra shows ratios between non-acetylated RG-I m/z=1574 (57%), mono acetylated RG-I m/z=1571 (36%) and diacetylated RG-I m/z=1568 (7%). **C)** MADLI-TOF MS spectra of RG-I DP6 after propionylation. Similarly, the ratio between non-acetylated to acetylated RG-I DP6 remains same (58% :35%:7%). Pro denotes propionyl groups and DAc denotes deuteroacetyl groups.

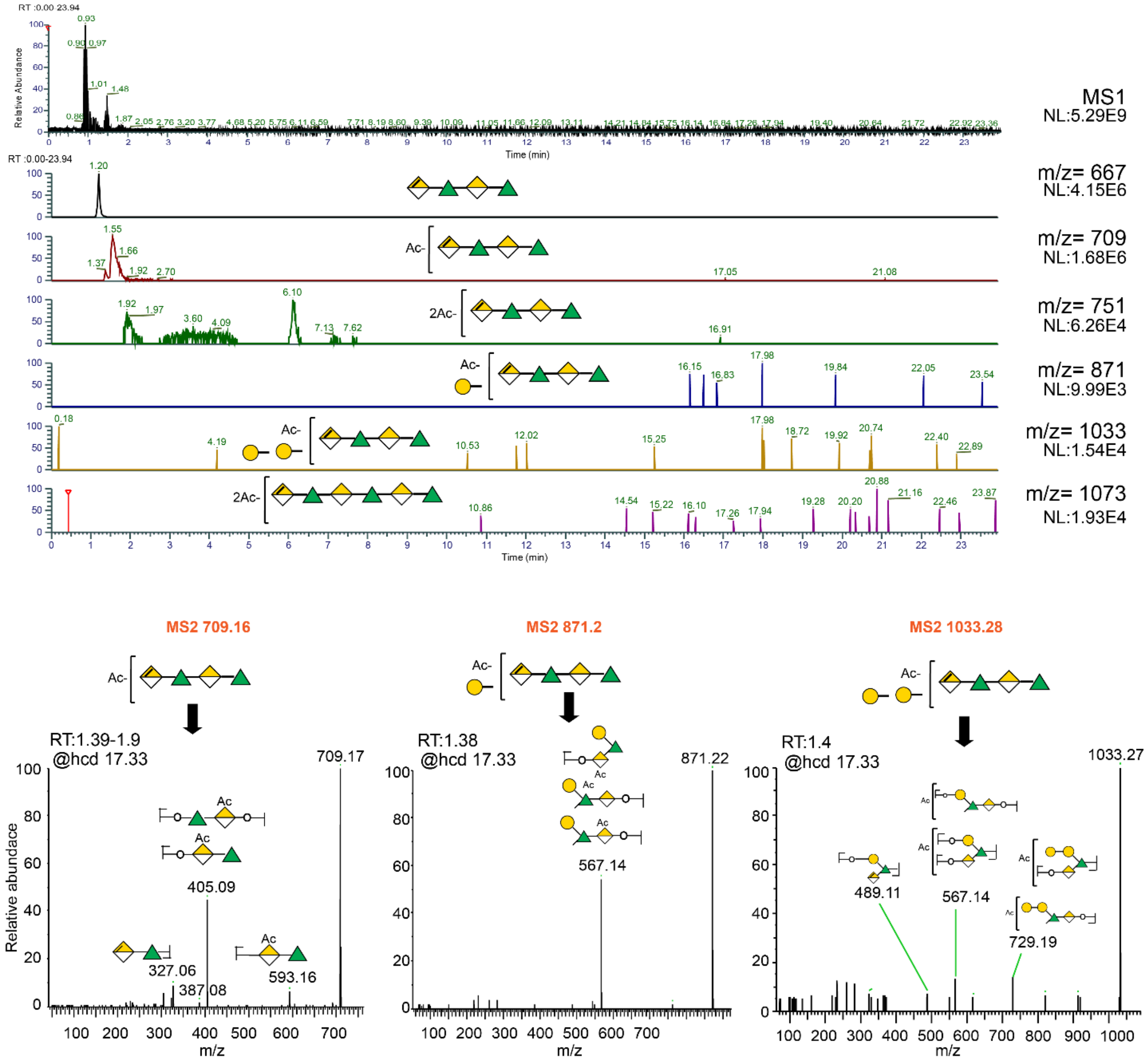

Supplementary Figure 12: LC elution profile and the LC-ESI-MS^2 spectra of the native (underivatized) celery RG-I oligomers in positive ion mode [M+Na]+; The top panel shows LC elution profile of the m/z= 709 mono acetylated RG-I DP4 and m/z= 871 mono acetylated RG-I with one galactose branching.

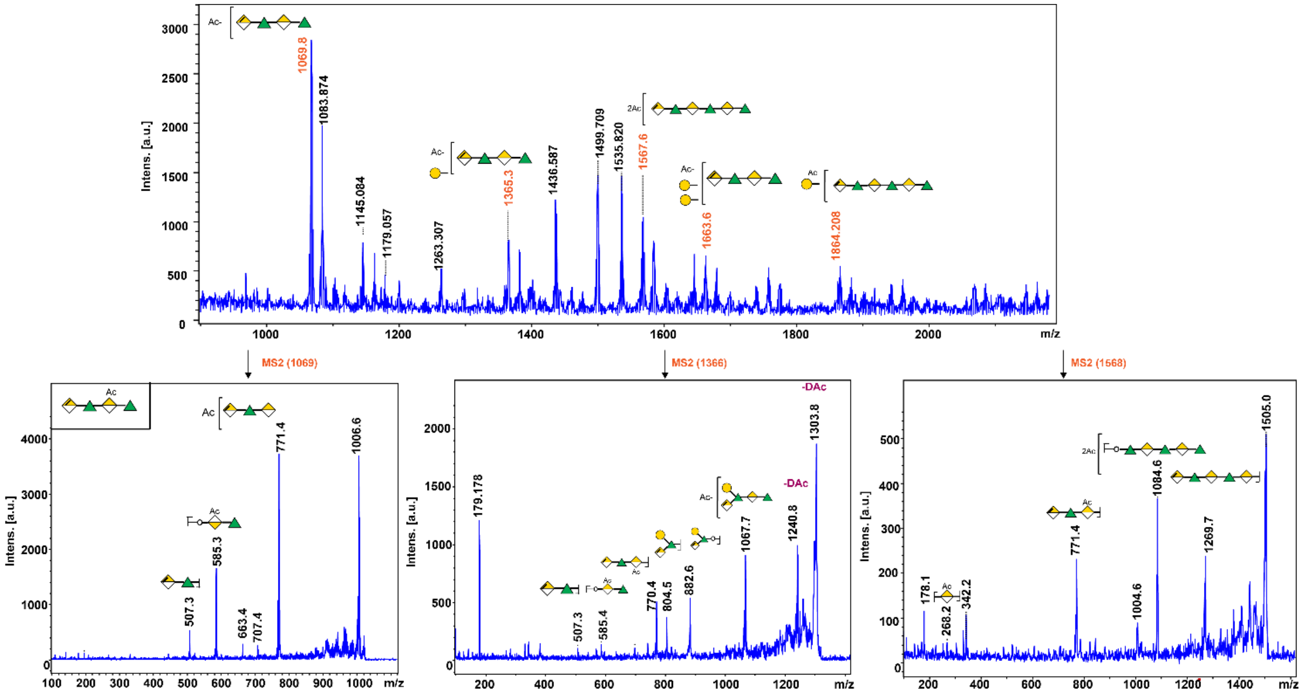

Supplementary Figure 13: MALDI-TOF MS spectra of deuteroacetylated oligomers derived from celery RG-I (top). The MS^2 spectra of different structures of RG-I; m/z =1069 mono acetylated RG-I DP4, m/z 1366 mono acetylated RG-I DP4 with galactose branching, m/z 1567 = diacetylated RG-I DP6 (bottom).

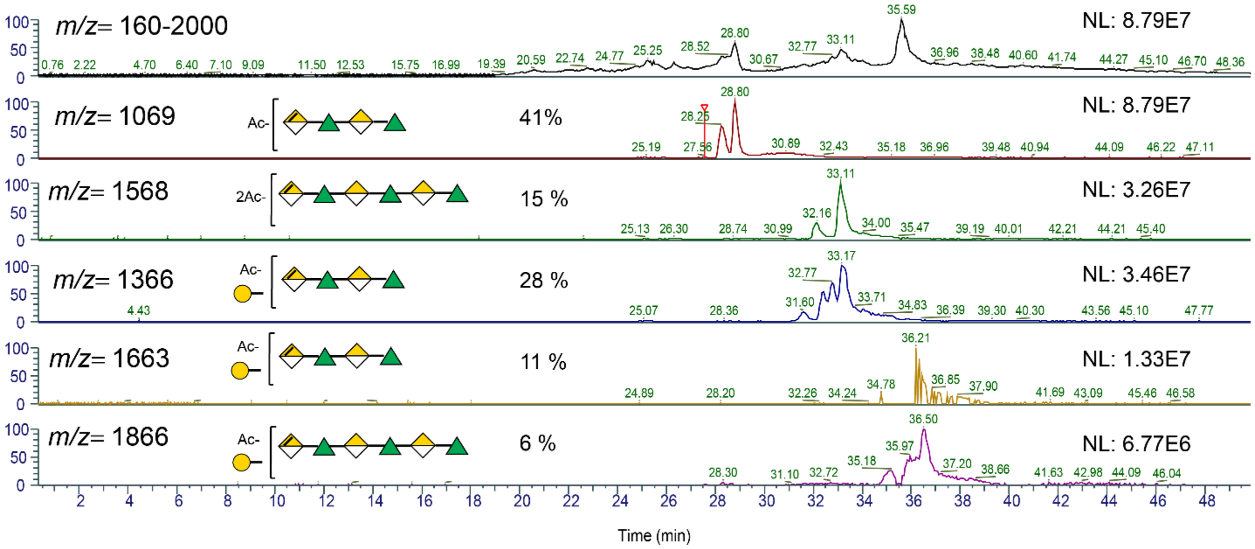

Supplementary Figure 14: Top: Full LC-MS acquisition of the mixture of deuteroacetylated oligomers derived from celery RG-I. Bottom: Extracted *m/z* values of different structural oligomers. Each structure shows different elution profile based on the degree of polymerization, degree of acetylation, branching pattern, and the length of the branching which related to the hydrophobicity of the oligomer. More hydrophobic the oligomer more time in the C18 reverse phase column and later elution. The ambiguous linkage positions of the acetyl group and the galactose reissue are shown in the brackets. The relative percentage of each structure shown in the spectra, calculated based on the peak intensity. NL: normalized intensity level.

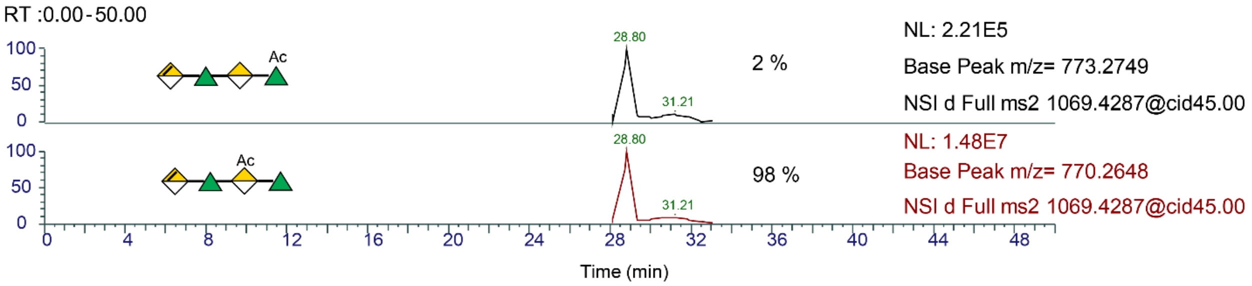

Supplementary Figure 15: LC Extraction of B_3 fragment; *m/z* =770 and *m/z* =773 derived from the MS2 of m/z= 1069 (RG-I_*O*AC DP4). The m/z=773 characteristic to the acetylation on the reducing-end rhamnose residue and the *m/z* =770 corresponds to the acetylation in the first GalA residue. The relative percentage of each isomer is shown to be calculated based on the relative intensity of the peaks.

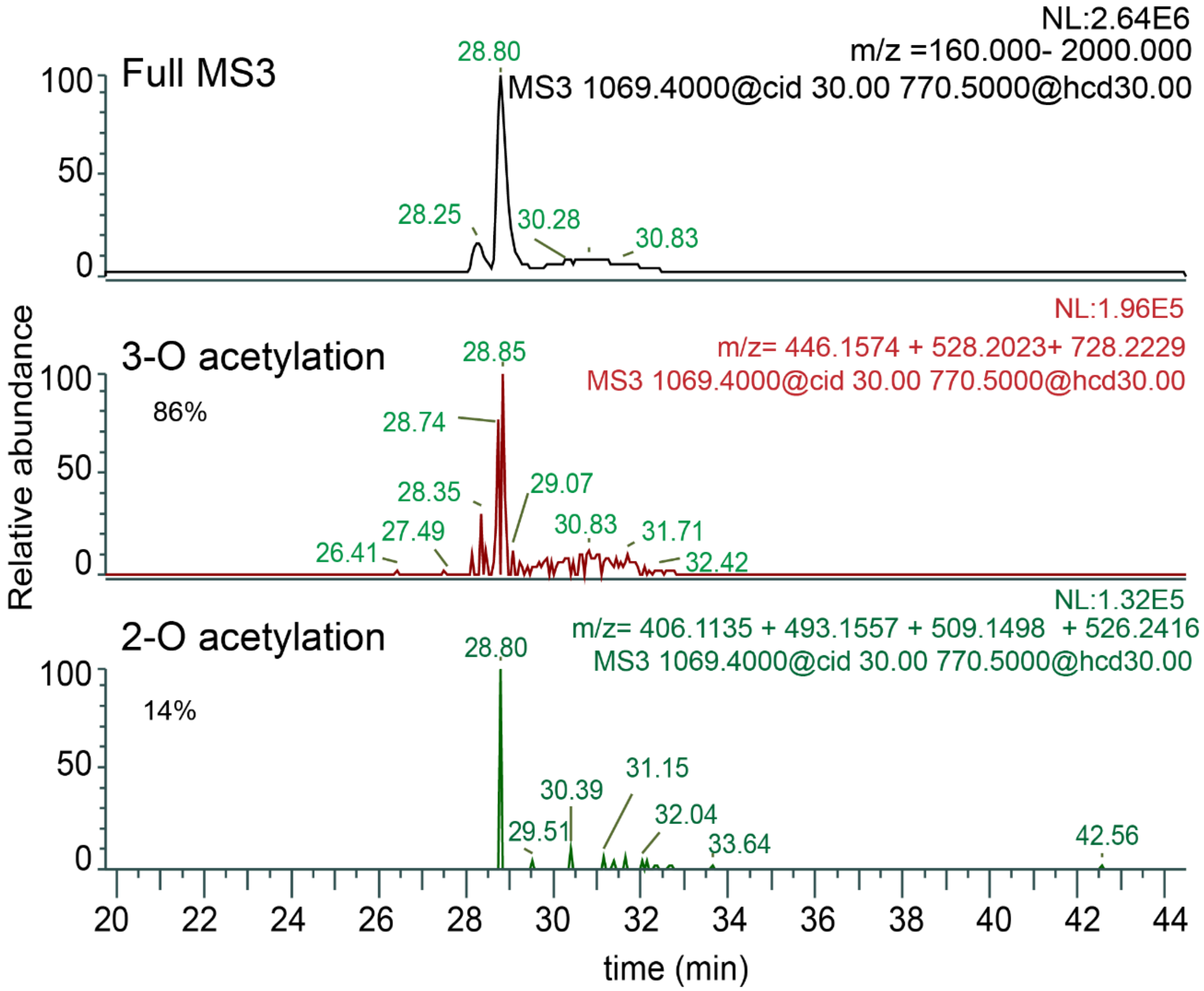

Supplementary Figure 16: Extracted *m/z* values for 3-*O*Ac and 2-*O*Ac from the full scan acquisition. The qualitative relative percentage of each structure shown in the spectra, calculated based on the peak intensities. Approximately the 3-*O*Ac is 86% and 2-OAc is 14%. NL: normalized intensity level.

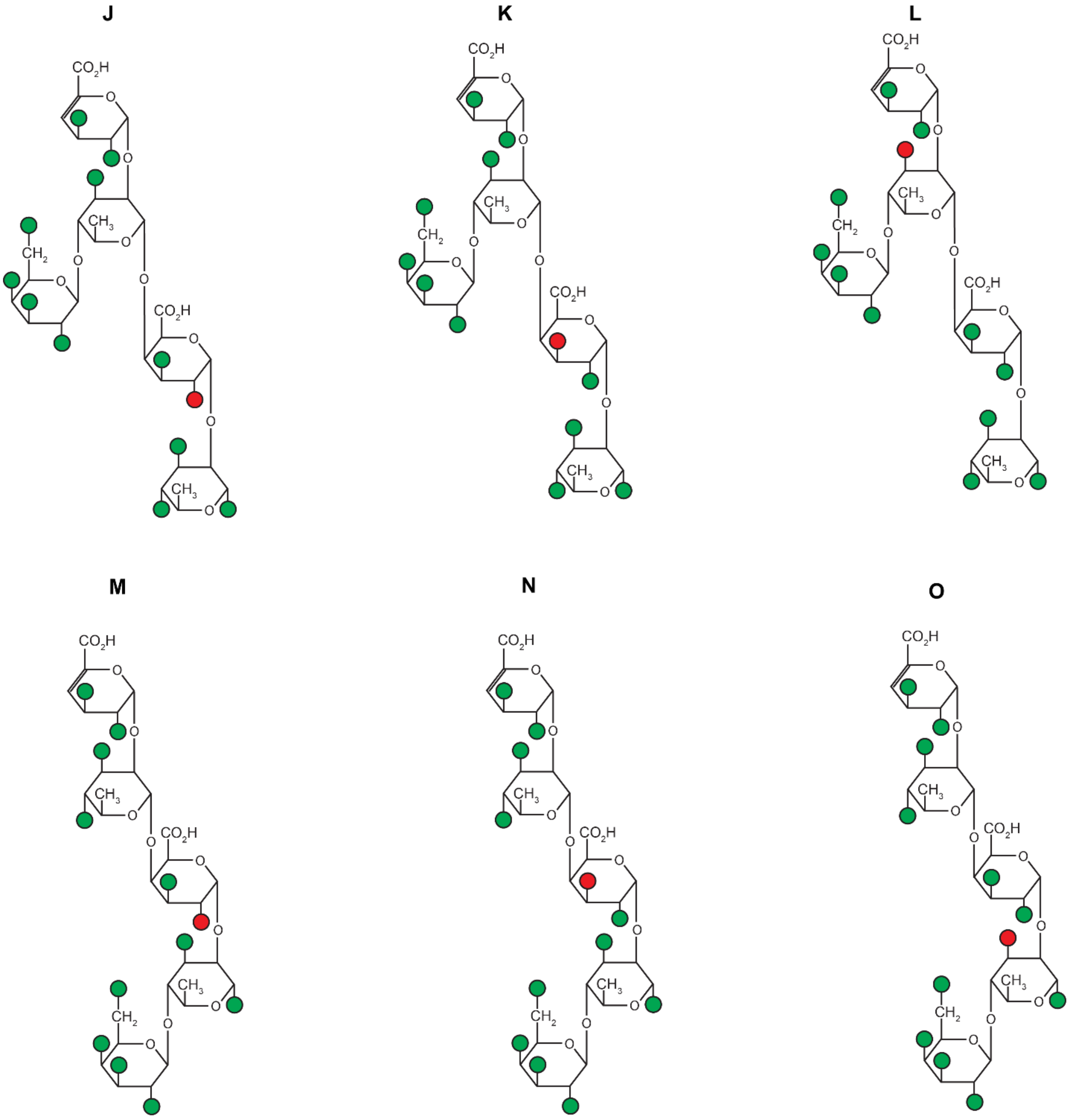

Supplementary Figure 17: The possible structures observed for the molecular ion *m/z*=1366; which comes from mono acetylated RG-I DP4 with one galactose branching. The structures are resulting from different acetylation position on the rhamnose and galacturonic acid, as well as the possible positions of the galactose residue. Based on the NMR the branching is on the rhamnose residue and deduce the possible structures.

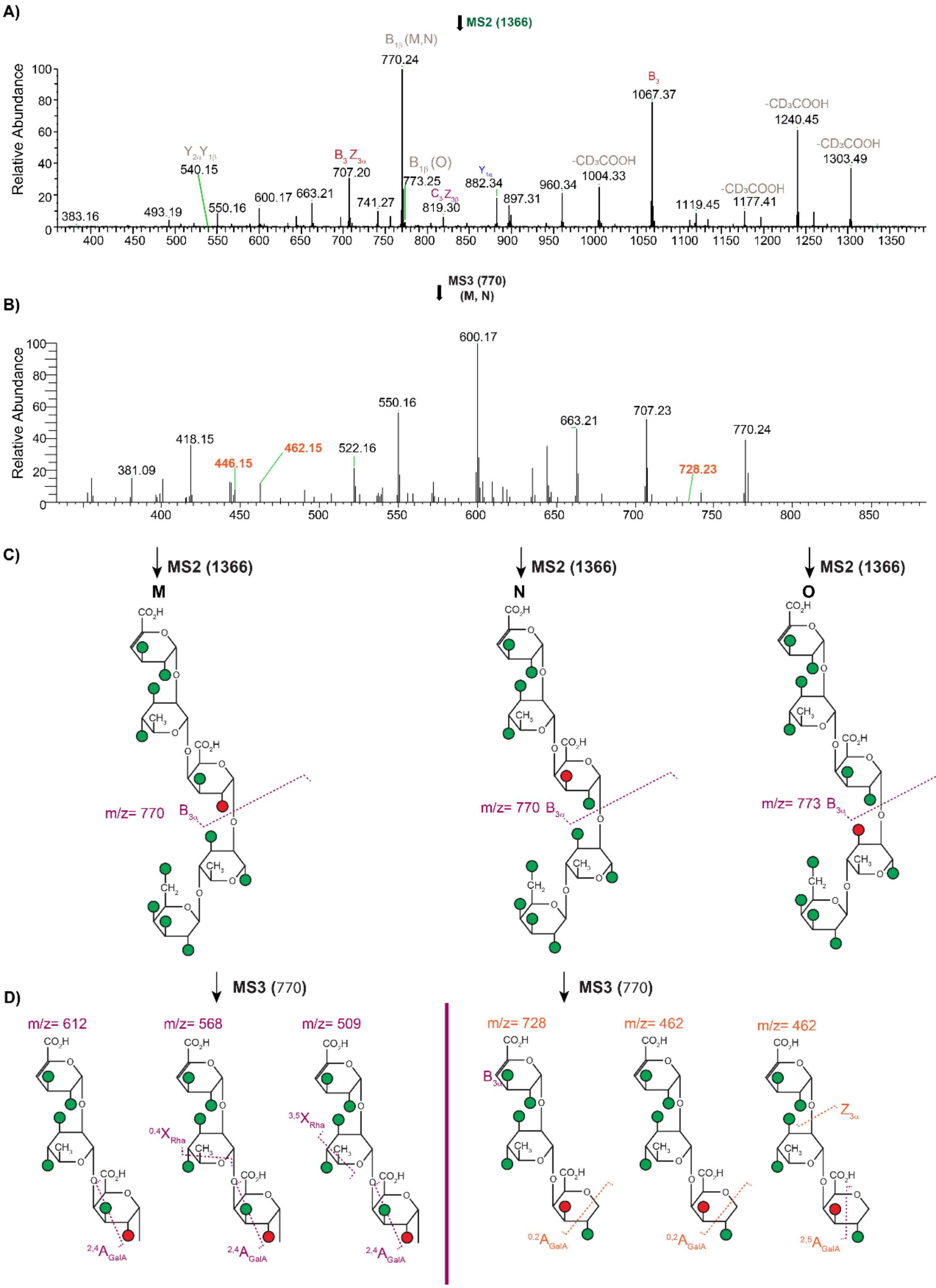

Supplementary Figure 18: **A)** MS^2 spectrum of *m/z*=1366 [M+Na]+ **B)** MS^3 spectrum of *m/z*= 770 (for M and N isomers) **C**) The M, N and O structures and cleavages observed in the MS spectra are labeled. The red circle denotes the *O*-acetyl groups, and the green circles are deuteroacetylated groups. **D)** Structure of the diagnostic cross-ring fragments observed in the MS^3 spectra for 2-*O*-acetylation structure M and 3-*O*-acetylation for structure N.

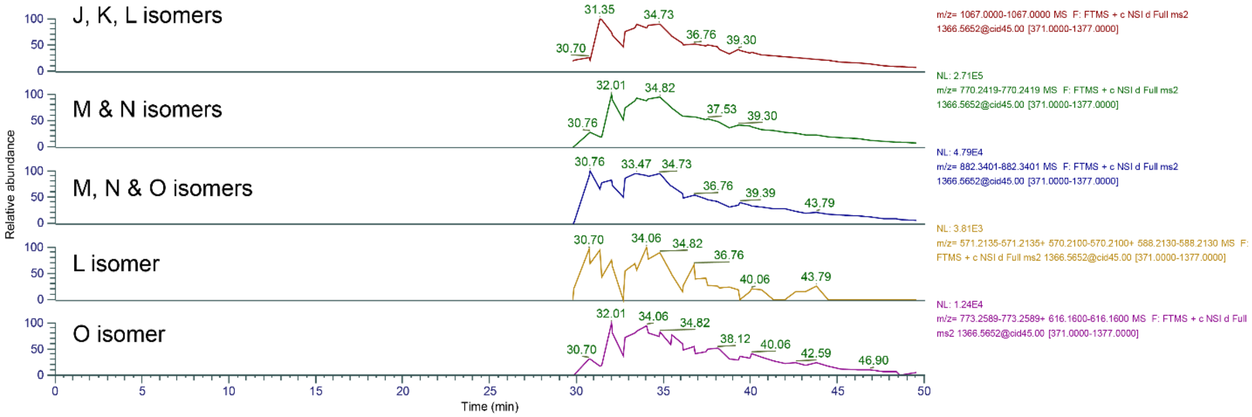

Supplementary Figure 19: Extraction of m/z values that are characteristic of different isomers from the full scan acquisition of MS^2 *m/z* =1366.

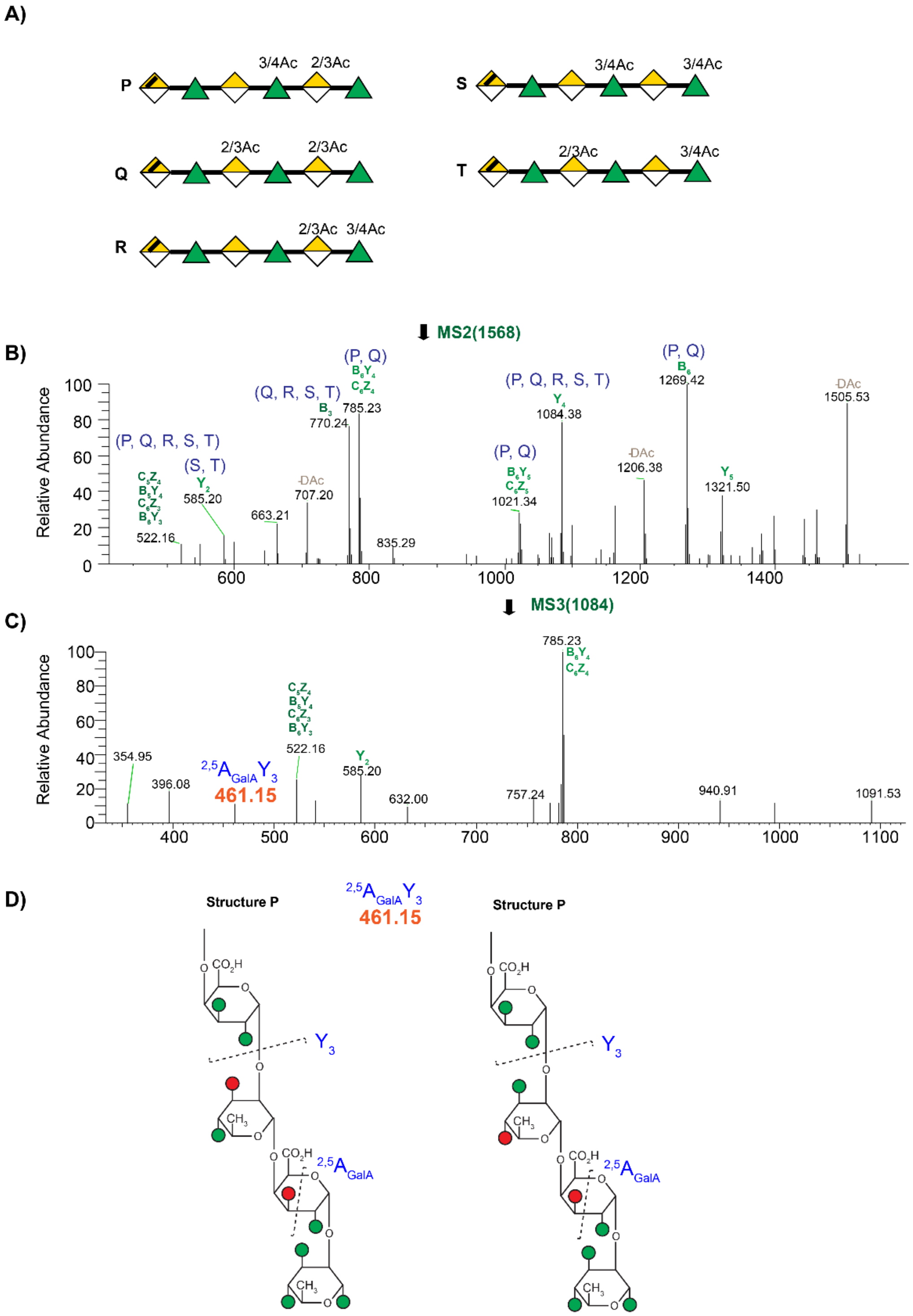

Supplementary Figure 20: A) Possible structures P, Q, R, S and T deduced from the MS^2 spectra of the m/z= 1568 **B)** MS^2 spectrum of *m/z*=1568 [M+Na]+, perdeuteroacytalated linear DP6 RG-I_2*O*Ac **C)** MS^3 spectrum of *m/z*= 1084) **D)** Diagnostic cross-ring cleavage observed in the MS spectra which pinpoints the GalA 3-*O*-acetylation on the structure P. The red circle denotes the acetyl groups, and the green circles are deuteroacetylated groups

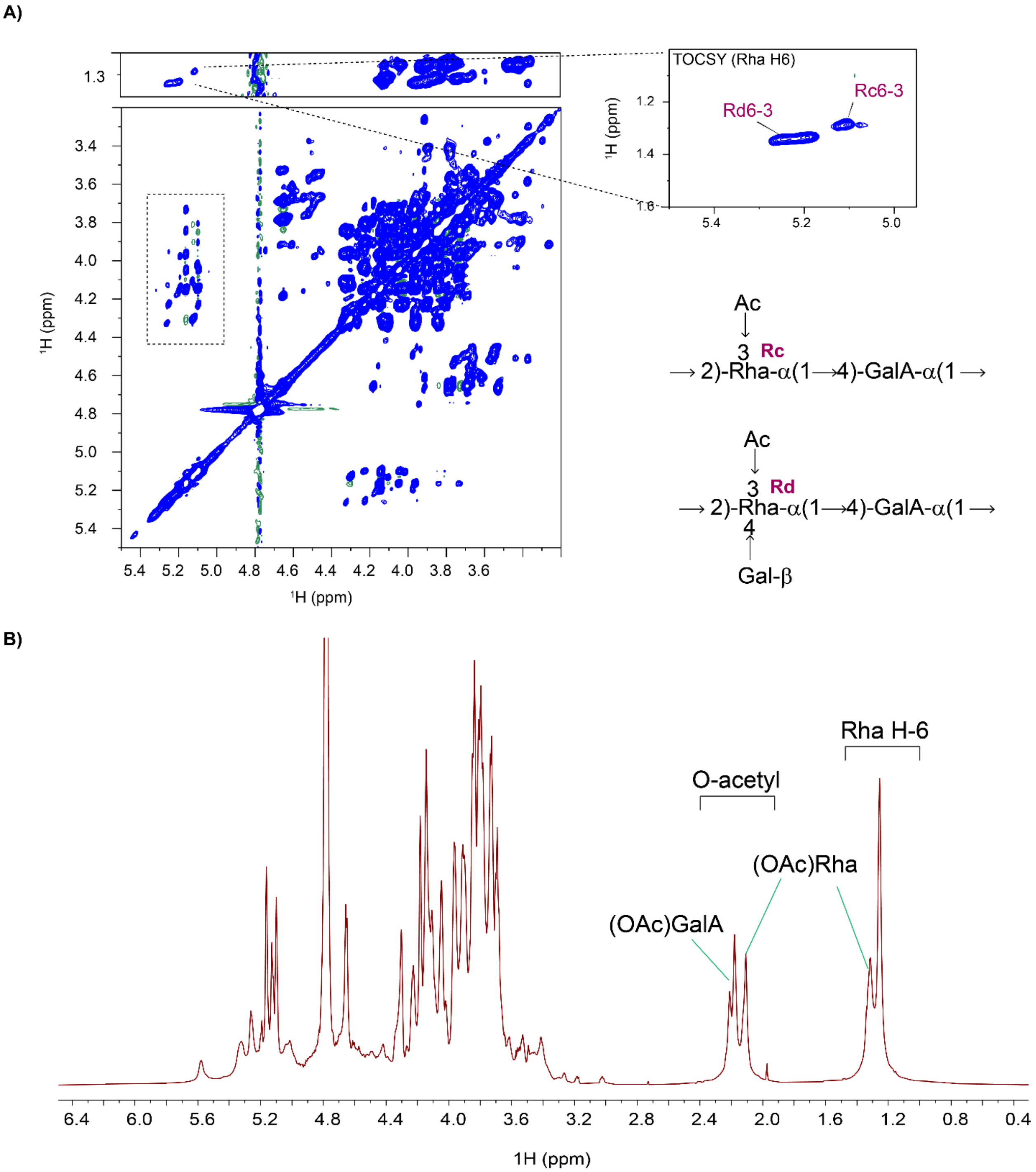

Supplementary Figure 21: A) ^{1}H -^{1}H 2D TOCSY spectrum of native RG-I celery (undigested). TOCSY allows the observation of correlations between the anomeric proton (H1) and all other protons (H2–H6) within the rhamnose C6 methyl group ($–CH_3$) which appears at ~1.3 ppm. These chemical shifts zoomed in shows the change in the position of the H6-H3 cross-peak compared to branched and non-branched acetylated rhamnose. **B)** 1D 1H NMR of native undigested celery RG-I. The 1D 1H NMR shows evidence of both rhamnose and GalA acetylation.[2, 3] Specific location of GalA acetylation cannot be made based on the NMR spectra.

Supplementary Table 1: NMR Acquisition Parameters

	Acquisition parameters at 25 °C at 800 MHz										
Sample	Experiments	d1 (s)	NS	td2	td1	aq2 (s)	aq1 (s)	sw2 (ppm)	sw1 (ppm)	Tm (ms)	Exp Time
RG-I OAc DP6	1D ^{1}H	1.5/ 30s	8	65536		2.03	-	20.2	-	-	38s/10 min
	COSY	1.5	4	2048	256	0.10	0.026	12	12	-	30min
	HSQC	1.5	16	2048	256	0.10	0.003	12	160	-	2hr
Celery RG-I	1D ^{1}H	1.0	4	65536	-	2.0	-	20.1	-	-	21s
	TOCSY	1.5	16	2048	256	0.13	0.05	9.7	3	120	2hr

Supplementary Table 2: ^{1}H and ^{13}C Chemical shifts (ppm) of RG-I samples.

RG-I_OAc DP6

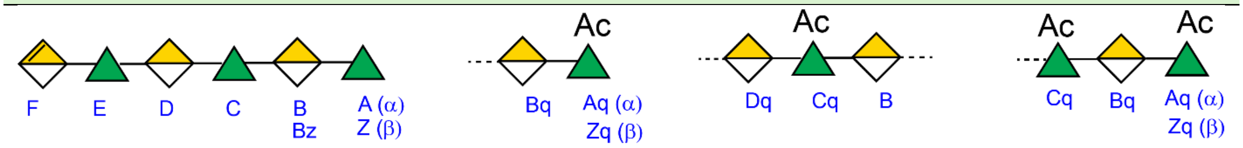

Residue	1	2	3	4	5	6
E	5.32<br>101.2	4.32<br>/	/	/	/	/
C	5.26<br>101.2	4.13<br>/	/	/	/	/
Cq	5.23<br>101.8	4.23<br>/	/	/	/	/
A(α)	5.22<br>94.32	3.98<br>/	/	/	/	/
Z (β)	4.93<br>96.6	3.99<br>/	/	/	/	/
Aq(α)	5.18<br>94.9	/	/	/	/	/
Zq(β)	5.00<br>96.2	/	/	/	/	/
F	5.11<br>100.5	3.81<br>/	/	/	/	/
B	5.05<br>100.4	3.93<br>/	/	/	/	/
D	5.01<br>100.1	3.94<br>/	/	/	/	/
Bq	4.97<br>101.9	3.91<br>/	/	/	/	/
Dq	4.96<br>101.7	3.92<br>/	/	/	/	/
Bz	5.16<br>103.7		/	/	/	/
**RG-I celery**						
Ac → 3 (Rc) →2)-Rha-α(1→4)-GalA-α(1→	/	/	5.11	3.59	/	1.28
Ac → 3 (Rd) →2)-Rha-α(1→4)-GalA-α(1→ with Gal-β → 4	/	/	5.19	3.94	/	1.33

/, not determined

Supplementary Table 3: Diagnostic cross-ring fragments observed in the MS^3 (m/z 770) $[M+Na]^+$ spectra for 3-*O*Ac and 2-*O*Ac.

Diagnostic cross Ring in MS2 (1069)-> MS3 (770)				
Acetyl position	Observed m/z	Theoretical m/z	|Δ mass difference|	Cleavage
3-*O*Ac	446.1674	446.1619	0.0055	$^{2,5}A_{GalA}Z$
	528.2123	528.2156	0.00329	$^{2,4}X_{GalA}$
	728.2329	728.2477	0.0148	$^{0,2}A_{GalA}$
	409.2206	409.1858	0.0348	$^{0,2}X_{GalA}$
2-*O*Ac	406.1235	406.1306	0.0071	$^{0,2}X_{GalA}$
	493.1657	493.1706	0.0049	$^{2,4}A_{GalA}{}^{0,3}X_{Rha}$
	509.1598	509.1655	0.0057	$^{2,4}A_{GalA}{}^{3,5}X_{Rha}$
	526.2516	526.2364	0.0152	$^{2,4}A_{GalA}{}^{0,3}X_{GalA}$
3*O*Ac/2*O*Ac	462.1627	462.1568	0.0059	$^{0,2}A_{GalA}Z$

Supplementary Table 4: Glycosidic cleavage fragments observed in the MS^2 spectra of m/z 1366 characteristics for different isomers. The green shading shows the cleavage observed for each isomer. m/z=1067, 819 are only for J, K and L isomers. m/z= 770 is only for M and N isomer.

MS2 (m/z= 1366)								
Observed mass [M+Na]+	Theoretical [M+Na]+ mass	Mass difference (Da)	J 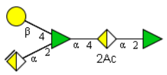	K 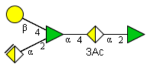	L 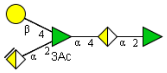	M 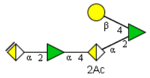	N 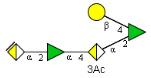	O 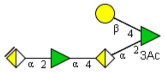
1067.3775	1067.39	0.0125	B_3	B_3	B_3			
882.3401	882.3589	0.0188				$Y_{2\alpha}$	$Y_{2\alpha}$	$Y_{2\alpha}$
819.301	819.3190	0.018	$B_3Y_{3\beta}/C_3Z_{3\beta}$	$B_3Y_{3\beta}/C_3Z_{3\beta}$	$B_3Y_{3\beta}/C_3Z_{3\beta}$			
770.2419	770.2583	0.0164				$B_{3\alpha}$	$B_{3\alpha}$	
773.2589	773.2771	0.0182						$B_{3\alpha}$
707.2030	707.2183	0.0153	B_3Z_3	B_3Z_3	B_3Z_3			
588.2133	588.2368	0.0235			Y_2			
571.2134	571.2387	0.0253			$C_2Y_{3\beta}$			
570.2119	570.2262	0.0143			Z_2			
540.1553	540.1885	0.0332				$Y_{2\alpha}$ $Y_{1\beta}$	$Y_{2\alpha}$ $Y_{1\beta}$	$Y_{2\alpha}$ $Y_{1\beta}$
585.2047	585.2179	0.0132	Y2	Y2				
556.2344	556.2469	0.0125	$C_2Z_{3\beta}$	$C_2Z_{3\beta}$				
567.1946	567.2074	0.0128	Z_{16}	Z_2				
574.3351	574.2575	0.0776	$C_2Y_{1\beta}$	$C_2Y_{1\beta}$				

Supplementary Table 5: Glycosidic cleavages and cross-ring fragments observed in the MS^3 spectra of m/z= 1067 and m/z= 770. The m/z=1067 shows diagnostic fragments for J, K and L isomers. The m/z=770 shows diagnostic fragments correspond to the M and N isomers. (x) denotes common cross ring fragments observed in two or more isomers. The diagnostic cleavages only present in the L isomer shaded in green.

Observed mass [M+Na]+	Theoretical mass [M+Na]+	Difference (Da)	J 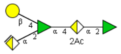	K 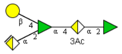	L 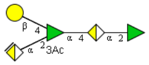
MS3=1067					
Glycosidic Cleavages					
383.1607	383.1702	0.0095	$C_{1\alpha}$	$C_{1\alpha}$	$C_{1\alpha}$
444.103	444.1463	0.0433	$B_2Z_{3\alpha}$	$B_2Z_{3\alpha}$	
480.127	480.1674	0.0404	$C_2Y_{3\,\alpha}$	$C_2Y_{3\,\alpha}$	
538.227	538.2364	0.0094	$B_2Z_{3\beta}$	$B_2\,Z_{3\beta}$	
553.256	553.2281	0.0279			$C_2Z_{3\beta}$ /$B_2Y_{3\beta}$
556.234	556.2469	0.0129	$C_2Z_{3\beta}$	$C_2Z_{3\beta}$	
571.206	571.2387	0.0327			$C_2Y_{3\beta}$
574.242	574.2575	0.0155	$C_3Y_{3\beta}$	$C_2Y_{3\beta}$	
804.308	804.3272	0.0192	B2	B2	
822.32	822.3378	0.0178	C2	C2	
Cross-ring cleavages					
355.1247	355.127	0.0023	x	x	x
357.1005	357.1063	0.0058	x	x	x
361.1968	361.1012	0.0956	x	x	x
363.1278	363.1169	0.0109	x	x	
364.1442	364.1201	0.0241	x		x
365.1496	365.1596	0.01	x	x	x
376.1233	376.1201	0.0032	x	x	
380.1386	380.1150	0.0236	x		
383.1607	383.1702	0.0095	x	x	x
391.1155	391.1118	0.0037	x	x	x
399.1086	399.1169	0.0083		x	x
403.1318	403.1482	0.0164	x	x	x
416.1925	416.1514	0.0411	x	x	
419.0376	419.1067	0.0691	x	x	x
423.191	423.2015	0.0105	$^{3,5}A_{Rha}$	$^{3,5}A_{Rha}$	$^{3,5}A_{Rha}$
491.112	491.1913	0.0793			$B^{0,4}X_{Rha}Z$
495.211	495.2226	0.0116			$^{2,5}A_{Rha}$
507.174	507.223	0.049			$^{1,5}A_{Rha}Z$
668.248	668.2630	0.015			$^{3,5}A_{GalA}Z$
717.296	717.2873	0.0087			$B^{1,3}X_{Rha}$
745.255	745.2822	0.0272			$^{1,4}A_{Rha}$
1012.36	1012.4126	0.0526	x		

Observed mass [M+Na]+	Theoretical mass [M+Na]+	Difference (Da)	M 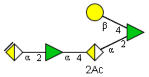	N 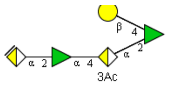
MS3=770				
Cross-ring cleavages				
728.2363	728.2477	0.0114		$^{0,2}A_{GalA}$
462.1456	462.1568	0.0112		$^{0,2}A_{GalA}Z$
446.1509	4461619	0.011		$^{2,5}A_{GalA}Z$
607.2245	607.2023	0.0222		$^{2,5}A_{GalA}\,^{1,3}X_{4uGalA}$
612.2025	612.2368	0.0343	$^{2,4}A_{GalA}$	
610.2092	610.2211	0.0119	$^{2,5}A_{GalA}\,^{1,3}X_{4uGalA}$	
568.2353	568.2105	0.0248	$^{2,4}A_{GalA}\,^{0,4}X_{Rha}$	
509.1412	509.1655	0.0243	$^{2,4}A_{GalA}\,^{3,5}X_{Rha}$	

References:

1. Domon, B.; Costello, C. E., A systematic nomenclature for carbohydrate fragmentations in FAB-MS/MS spectra of glycoconjugates. *Glycoconjugate J.* **1988,** *5*, 397-409.

2. Zhang, L.; Vlach, J.; Black, I. M.; Archer-Hartmann, S.; Heiss, C.; Azadi, P.; Urbanowicz, B. R., The pectin puzzle: Decoding the fine structure of rhamnogalacturonan-I (RG-I) in Arabidopsis thaliana uncovers new pectin features. *Carbohydr. Polym.* **2025,** *368*, 124161.

3. Perrone, P.; Hewage, C. M.; Thomson, A. R.; Bailey, K.; Sadler, I. H.; Fry, S. C., Patterns of methyl and O-acetyl esterification in spinach pectins: new complexity. *Phytochemistry* **2002,** *60* (1), 67-77.
